# Supplementary material for: COVID-19 and pregnancy: An umbrella review of clinical presentation, vertical transmission, and maternal and perinatal outcomes
Source: PLoS One. 2021 Jun 29;16(6):e0253974. doi: 10.1371/journal.pone.0253974 (PMC8241118; doi:10.1371/journal.pone.0253974)
Supplement: S3 File — (DOCX) [file pone.0253974.s003.docx]

# S3 File. Search strategy

**PubMed**

**Search Query**

#53  #20 AND #51 AND #52

#52  (Systematic Review[sb] OR Systematic Review[tiab] OR Meta-Analysis[pt] OR Meta-Analys*[tiab] OR "Cochrane Database Syst Rev"[ta] OR Metaanalysis[tiab] OR Metanalysis[tiab] OR Overview[ti] OR (Review[ti] AND Literature[ti]) OR (MEDLINE[tiab] AND Cochrane[tiab]))

#51  #21 OR #22 OR #23 OR #24 OR #25 OR #26 OR #27 OR #28 OR #29 OR #30 OR #31 OR #32 OR #33 OR #34 OR #35 OR #36 OR #37 OR #38 OR #39 OR #40 OR #41 OR #42 OR #43 OR #44 OR #45 OR #46 OR #47 OR #48 OR #49 OR #50

#50  Miscarriage*[tiab]

#49  Abortion*[tiab]

#48  Abortion, Spontaneous[Mesh]

#47  Partum[tiab]

#46  Parturition*[tiab]

#45  Childbirth*[tiab]

#44  Parturition[Mesh]

#43  Gestational[tiab]

#42  Perinatal[tiab]

#41  Perinatal Care[Mesh]

#40  Mother*[tiab]

#39  Mothers[Mesh]

#38  Intrauterine[tiab]

#37  Vertical[tiab]

#36  Mother-to-Child[tiab]

#35  Infectious Disease Transmission, Vertical[Mesh]

#34  Fetomaternal[tiab]

#33  Materno Fetal[tiab]

#32  Maternofetal[tiab]

#31  Fetus[tiab]

#30  Fetal[tiab]

#29  Fetus[Mesh]

#28  Pregnan*[tiab]

#27  Pregnancy Complications, Infectious[Mesh]

#26  Pregnancy[Mesh]

#25  Ante-Natal[tiab]

#24  Antenatal[tiab]

#23  Pre-Natal Care*[tiab]

#22  Prenatal Care*[tiab]

#21  Prenatal Care[Mesh]

#20  #1 OR #2 OR #3 OR #4 OR #5 OR #6 OR #7 OR #8 OR #9 OR #10 OR #11 OR #12 OR #13 OR #14 OR #15 OR #16 OR #17 OR #18 OR #19

#19  N Cov[tiab]

#18  CV-19[tiab]

#17  CV19*[tiab]

#16  HCov*[tiab]

#15  Corono Virus[tiab]

#14  Virus Corona[tiab]

#13  Coronovir*[tiab]

#12  Coronavir*[tiab]

#11  Pneumonia[tiab] AND Wuhan[tiab] AND 2019[tiab]

#10  SARS-CoV2[tiab]

#9  SARS-CoV-2[tiab]

#8  2019-nCoV[tiab]

#7  COVID19*[tiab]

#6  COVID-19[tiab]

#5  Corona Virus[tiab]

#4  COVID-19[Supplementary Concept]

#3  Severe Acute Respiratory Syndrome Coronavirus 2[Supplementary Concept]

#2  Spike protein, SARS-CoV-2 [Supplementary Concept]

#1  Coronavirus[Mesh]

**Cochrane Library (Wiley)**

ID Search

#1 MeSH descriptor: [Coronavirus] explode all trees

#2 (Corona NEAR/1 Virus):ti,ab,kw

#3 COVID-19:ti,ab,kw

#4 COVID19*:ti,ab,kw

#5 "2019-nCoV":ti,ab,kw

#6 "SARS-CoV-2":ti,ab,kw

#7 SARS-CoV2:ti,ab,kw

#8 (Pneumonia AND Wuhan AND 2019):ti,ab,kw

#9 Coronavir*:ti,ab,kw

#10 Coronovir*:ti,ab,kw

#11 (Corono NEAR/1 Virus):ti,ab,kw

#12 HCov*:ti,ab,kw

#13 CV19*:ti,ab,kw

#14 CV-19:ti,ab,kw

#15 N-Cov:ti,ab,kw

#16 #1 OR #2 OR #3 OR #4 OR #5 OR #6 OR #7 OR #8 OR #9 OR #10 OR #11 OR #12 OR #13 OR #14 OR #15

#17 MeSH descriptor: [Prenatal Care] explode all trees

#18 (Prenatal NEAR/1 Care*):ti,ab,kw

#19 (Pre-Natal NEAR/1 Care*):ti,ab,kw

#20 Antenatal:ti,ab,kw

#21 Ante-Natal:ti,ab,kw

#22 MeSH descriptor: [Pregnancy] explode all trees

#23 MeSH descriptor: [Pregnancy Complications, Infectious] explode all trees

#24 Pregnan*:ti,ab,kw

#25 MeSH descriptor: [Fetus] explode all trees

#26 Fetal:ti,ab,kw

#27 Fetus:ti,ab,kw

#28 Maternofetal:ti,ab,kw

#29 (Materno NEAR/2 Fetal):ti,ab,kw

#30 Fetomaternal:ti,ab,kw

#31 MeSH descriptor: [Infectious Disease Transmission, Vertical] explode all trees

#32 "Mother-to-Child":ti,ab,kw

#33 Vertical:ti,ab,kw

#34 Intrauterine:ti,ab,kw

#35 MeSH descriptor: [Mothers] explode all trees

#36 Mother*:ti,ab,kw

#37 MeSH descriptor: [Perinatal Care] explode all trees

#38 Perinatal:ti,ab,kw

#39 Gestational:ti,ab,kw

#40 MeSH descriptor: [Parturition] explode all trees

#41 Childbirth*:ti,ab,kw

#42 Parturition*:ti,ab,kw

#43 Partum:ti,ab,kw

#44 MeSH descriptor: [Abortion, Spontaneous] explode all trees

#45 Abortion*:ti,ab,kw

#46 Miscarriage*:ti,ab,kw

#47 #17 OR #18 OR #19 OR #20 OR #21 OR #22 OR #23 OR #24 OR #25 OR #26 OR #27 OR #28 OR #29 OR #30 OR #31 OR #32 OR #33 OR #34 OR #35 OR #36 OR #37 OR #38 OR #39 OR #40 OR #41 OR #42 OR #43 OR #44 OR #45 OR #46

#48 #16 AND #47 in Cochrane Reviews

**EMBase (Elsevier)**

No. Query

#50. #48 AND #

#49. 'systematic review':ti,ab OR 'meta análisis (topic)':pt OR 'meta analysis':ti,ab OR cochrane:jt OR metaanalysis:ti,ab OR metanalysis.ti,ab. OR (medline:ab AND cochrane:ab) OR overview:ti OR (review:ti AND literature:ti)

#48. #17 AND #47

#47. #18 OR #19 OR #20 OR #21 OR #22 OR #23 OR #24 OR #25 OR #26 OR #27 OR #28 OR #29 OR #30 OR #31 OR #32 OR #33 OR #34 OR #35 OR #36 OR #37 OR #38 OR #39 OR #40 OR #41 OR #42 OR #43 OR #44 OR #45 OR #46

#46. miscarriage*:ti,ab

#45. abortion*:ti,ab

#44. 'spontaneous abortion'/exp

#43. partum:ti,ab

#42. parturition*:ti,ab

#41. childbirth*:ti,ab

#40. 'birth'/exp

#39. gestational:ti,ab

#38. perinatal:ti,ab

#37. 'perinatal care'/exp

#36. mother*:ti,ab

#35. 'mother'/exp

#34. intrauterine:ti,ab

#33. vertical:ti,ab 1

#32. 'mother-to-child':ti,ab

#31. 'vertical transmission'/exp

#30. fetomaternal:ti,ab

#29. (materno NEAR/1 fetal):ti,ab

#28. maternofetal:ti,ab

#27. fetus:ti,ab

#26. fetal:ti,ab

#25. 'fetus'/exp

#24. pregnan*:ti,ab

#23. 'pregnancy'/exp

#22. 'ante natal':ti,ab

#21. antenatal:ti,ab

#20. ('pre natal' NEAR/1 care*):ti,ab

#19. (prenatal NEAR/1 care*):ti,ab

#18. 'prenatal care'/exp

#17. #1 OR #2 OR #3 OR #4 OR #5 OR #6 OR #7 OR #8 OR #9 OR #10 OR #11 OR #12 OR #13 OR #14 OR #15 OR #16

#16. 'n cov':ti,ab

#15. 'cv 19':ti,ab

#14. cv19*:ti,ab

#13. hcov*:ti,ab

#12. (corono NEAR/1 virus):ti,ab

#11. coronovir*:ti,ab

#10. coronavir*:ti,ab

#9. pneumonia:ti,ab AND wuhan:ti,ab AND 2019:ti,ab

#8. 'sars cov2':ti,ab

#7. 'sars cov 2':ti,ab

#6. '2019 ncov':ti,ab

#5. covid19*:ti,ab

#4. 'covid 19':ti,ab

#3. (corona NEAR/1 virus):ti,ab

#2. 'coronavirus disease 2019'/exp

#1. 'coronavirinae'/exp

**LILACS**

(MH Coronavirus OR COVID-19 OR COVID19$ OR Corona OR Corono OR 2019-nCoV OR SARS-CoV-2 OR SARS-CoV2 OR Coronavir$ OR Coronovir$ OR HCov$ OR CV19$ OR CV-19 OR N-Cov) AND (MH Prenatal Care OR Prenatal-Care$ OR Pre-Natal-Care$ OR Antenatal OR Ante-Natal OR MH Pregnancy OR MH Pregnancy Complications, Infectious OR Pregnan$ OR Embarazo$ OR Gravid$ OR MH Fetus OR Feto OR Fetus OR Fetal OR Maternofetal OR Materno-Fetal OR Fetomaternal OR MH Infectious Disease Transmission, Vertical OR Mother-to-Child OR Vertical OR Intrauterin$ OR MH Mothers OR Mother$ OR Madre$ OR Maes OR Mae OR MH Perinatal Care OR Perinatal OR Gestational OR Gestacional OR MH Parturition OR Childbirth$ OR Parto OR Partum OR MH Abortion, Spontaneous OR Abortion OR Aborto OR Miscarriage$) AND (MH Metanálisis como Asunto OR PT metanalisis OR Meta-Anális$ OR Metaanális$ OR metanalis$ OR ((revisión OR Review OR Revisao) AND (sistematic$ OR Systematic)) ) [Words]

**Web of Science (SCI-EXPANDED, SSCI, A&HCI, ESCI)**

# 51 #50 AND #49

# 50 TS=Systematic Review OR TI=Systematic Review OR TS=Meta-Analysis OR TI=Meta-Analys* OR TI=Metaanalysis OR TI=Metanalysis OR TI=Overview OR SO=(COCHRANE DATABASE OF SYSTEMATIC REVIEWS)

# 49 #48 AND #17

# 48 #18 OR #19 OR #20 OR #21 OR #22 OR #23 OR #24 OR #25 OR #26 OR #27 OR #28 OR #29 OR #30 OR #31 OR #32 OR #33 OR #34 OR #35 OR #36 OR #37 OR #38 OR #39 OR #40 OR #41 OR #42 OR #43 OR #44 OR #45 OR #46 OR #47

# 47 TI=Miscarriage*

# 46 TI=Abortion*

# 45 TS=Abortion, Spontaneous

# 44 TI=Partum

# 43 TI=Parturition*

# 42 TI=Childbirth*

# 41 TS=Parturition

# 40 TI=Gestational

# 39 TI=Perinatal

# 38 TS=Perinatal Care

# 37 TI=Mother*

# 36 TS=Mothers

# 35 TI=Intrauterine

# 34 TI=Vertical

# 33 TI=Mother-to-Child

# 32 TS=Infectious Disease Transmission, Vertical

# 31 TI=Fetomaternal

# 30 TI=Materno Fetal

# 29 TI=Maternofetal

# 28 TI=Fetus

# 27 TI=Fetal

# 26 TS=Fetus

# 25 TI=Pregnan*

# 24 TS=Pregnancy Complications, Infectious

# 23 TS=Pregnancy

# 22 TI=Ante-Natal

# 21 TI=Antenatal

# 20 TI=Pre-Natal Care*

# 19 TI=Prenatal Care*

# 18 TS=Prenatal Care

# 17 #1 OR #2 OR #3 OR #4 OR #5 OR #6 OR #7 OR #8 OR #9 OR #10 OR #11 OR #12 OR #13 OR #14 OR #15 OR #16

# 16 TI=N Cov

# 15 TI=CV-19

# 14 TI=CV19*

# 13 TI=HCov*

# 12 TI=Corono Virus

# 11 TI=Virus Corona

# 10 TI=Coronovir*

# 9 TI=Coronavir*

# 8 TI=SARS-CoV2

# 7 TI=SARS-CoV-2

# 6 TI=2019-nCoV

# 5 TI=COVID19*

# 4 TI=Corona Virus

# 3 TI=COVID-19

# 2 TS=COVID-19

# 1 TS=Corona Virus

**CNKI (ENG** **(E) Medicine & Public Health**)

TI=Covid-19 OR TI=2019-nCoV OR TI=SARS-CoV-2 OR TI=SARS-CoV2 OR TI=Coronavirus OR TI=Covid19) AND (TI=Prenatal OR TI=Antenatal OR TI=Perinatal OR TI=Pregnancy OR TI=Fetal OR TI=Maternofetal OR TI=Fetomaternal OR TI=Vertical OR TI=Mother OR TI=Maternal OR TI=Childbirth OR TI=Partum OR TI=Parturition OR TI=Abortion OR TI=Miscarriage)

**LOVE**

Prenatal Care* OR Pre-Natal Care* OR Antenatal OR Ante-Natal OR Pregnan* OR Fetal OR Fetus OR Maternofetal OR Materno Fetal OR Fetomaternal OR Mother-to-Child OR Vertical OR Intrauterine OR Mother* OR Perinatal OR Gestational OR Childbirth* OR Parturition* OR Partum OR Abortion* OR Miscarriage*) Filter results: Systematic Review
