# Supplementary material for: COVID-19 and pregnancy: An umbrella review of clinical presentation, vertical transmission, and maternal and perinatal outcomes
Source: PLoS One. 2021 Jun 29;16(6):e0253974. doi: 10.1371/journal.pone.0253974 (PMC8241118; doi:10.1371/journal.pone.0253974)
Supplement: S4 File — (DOCX) [file pone.0253974.s004.docx]

# S4 File. Excluded studies & exclusion reasons

| Authors (2020) | Title of the study | Exclusion reason |
| --- | --- | --- |
| Ashary[1] | Single-Cell RNA-seq Identifies Cell Subsets in Human Placenta That Highly Expresses Factors Driving Pathogenesis of SARS-CoV-2 | Wrong outcomes |
| Azarkish[2] | Impact of COVID-19 infection on maternal and neonatal outcomes: a review of 287 pregnancies | Unmet DARE definition of SR |
| Boyadzhieva[3] | Coronavirus disease 2019 (COVID-19) during pregnancy in patients with rheumatic diseases | Unmet DARE definition of SR |
| Brown[4] | Perinatal mental health and the COVID-19 pandemic | Unmet DARE definition of SR |
| Cabero-Pérez[5] | [Infection by SARS-CoV-2 in pregnancy and possibility of transmission to neonates: A systematic revision] | Unmet DARE definition of SR |
| Campos[6] | Increasing maternal mortality associated with COVID-19 and shortage of intensive care is a serious concern in low‐resource settings | Unmet DARE definition of SR |
| Capobianco[7] | COVID-19 in pregnant women: A systematic review and meta-analysis | Unmet DARE definition of SR |
| Chamseddine[8] | Pregnancy and Neonatal Outcomes in SARS-CoV-2 Infection: a systematic review | Unmet DARE definition of SR |
| Chang[9] | Clinical characteristics and diagnostic challenges of pediatric COVID-19: A systematic review and meta-analysis | Wrong outcomes |
| Dawei[10] | Asymptomatic COVID-19 infection in late pregnancy indicated no vertical transmission | Unmet DARE definition of SR |
| Delfino[11] | SARS‐CoV‐2 possible contamination of genital area: implications for sexual and vertical transmission routes | Unmet DARE definition of SR |
| Delgado[12] | Rates of Maternal and Perinatal Mortality and Vertical Transmission in Pregnancies Complicated by Severe Acute Respiratory Syndrome Coronavirus 2 (SARS-Co-V-2) Infection: A Systematic Review | Unmet DARE definition of SR |
| Di Mascio[13] | Outcome of coronavirus spectrum infections (SARS, MERS, COVID-19) during pregnancy: a systematic review and meta-analysis | Duplicated |
| Di Nardo[14] | A literature review of 2019 novel coronavirus (SARS-CoV2) infection in neonates and children | Unmet DARE definition of SR |
| Dubey[15] | Maternal and neonatal characteristics and outcomes among COVID-19 infected women: An updated systematic review and meta-analysis | Unmet DARE definition of SR |
| Duran[16] | COVID-19 and newborn health: systematic review | Duplicated |
| Elshafeey[17] | A systematic scoping review of COVID-19 during pregnancy and childbirth | Unmet DARE definition of SR |
| Furlan[18] | A Systematic Review of Pregnancy and Coronavirus Infection: Maternal, Fetal and Neonatal Outcomes | Duplicated |
| Galang[19] | Severe Coronavirus Infections in Pregnancy: A Systematic Review | Unmet DARE definition of SR |
| Gillian[20] | Clinical update on COVID-19 in pregnancy: A review article | Unmet DARE definition of SR |
| Hoekstra[21] | Corticosteroid use in COVID-19 patients: A systematic review and meta-analysis on clinical outcomes | Wrong population |
| Ibrahim[22] | Review of published systematic reviews and meta-analyses on COVID-19 | Wrong population |
| IETSI[23] | [SARS-COV-2 mother-fetus transmission: rapid synthesis of evidence] | Unmet DARE definition of SR |
| Irani[24] | Novel coronavirus disease 2019 and perinatal outcomes | Unmet DARE definition of SR |
| Jahangir[25] | Clinical manifestations and outcomes of COVID-19 in the paediatric population: a systematic review | Unmet DARE definition of SR |
| Juan[26] | Effects of Coronavirus Disease 2019 (COVID-19) on Maternal, Perinatal and Neonatal Outcomes: a Systematic Review of 266 Pregnancies | Duplicated |
| Juan[27] | COVID-19/SARS-CoV-2 News from Preprints; Effects of Coronavirus Disease 2019 (COVID-19) on Maternal, Perinatal and Neonatal Outcomes: a Systematic Review of 266 Pregnancies | Unmet DARE definition of SR |
| Kahathuduwa[28] | Case fatality rate in COVID-19: a systematic review and meta-analysis | Wrong outcomes |
| Khalil[29] | Coronavirus - COVID-19; Findings from A. Khalil and Co-Researchers Advance Knowledge in COVID-19 Outcome of Coronavirus spectrum infections (SARS, MERS, COVID 1 -19) during pregnancy: a systematic review and meta-analysis | Unmet DARE definition of SR |
| Khalili[30] | Male Fertility and the COVID-19 Pandemic: Systematic Review of the Literature | Unmet DARE definition of SR |
| Klaritsch[31] | COVID-19 During Pregnancy and Puerperium - A Review by the Austrian Society of Gynaecology and Obstetrics (OEGGG) | Unmet DARE definition of SR |
| Lubbe[32] | Breastfeeding during the COVID-19 pandemic - a literature review for clinical practice | Unmet DARE definition of SR |
| Mark[33] | Community-Onset SARS-CoV-2 Infection in Young Infants: A Systematic Review | Unmet DARE definition of SR |
| Martins-Filho[34] | [To breastfeed or not to breastfeed? Lack of evidence on the presence of SARS-CoV-2 in breastmilk of pregnant women with COVID-19] | Duplicated |
| Mascarenhas[35] | COVID-19 and the production of knowledge regarding recommendations during pregnancy: a scoping review | Unmet DARE definition of SR |
| Melekhina[36] | Clinical characteristics of covid-19 in children of different ages. Literature review as of april 2020 | Unmet DARE definition of SR |
| Mojgan[37] | Vertical Transmission of Coronavirus Disease 19 (COVID-19) from Infected Pregnant Mothers to Neonates: A Review | Unmet DARE definition of SR |
| Mullins[38] | Coronavirus in pregnancy and delivery: rapid review | Unmet DARE definition of SR |
| Mullins[39] | COVID-19/SARS-CoV-2 News from Preprints; Coronavirus in Pregnancy and Delivery: Rapid Review and Expert Consensus (Published March 8, 2020) | Unmet DARE definition of SR |
| Mullins[40] | Coronavirus - COVID-19; Recent Findings from North Bristol NHS Trust Has Provided New Data on COVID-19 (Coronavirus in pregnancy and delivery: rapid review) | Unmet DARE definition of SR |
| Oskovi-Kaplan[41] | Coronavirus - COVID-19; New Findings from Maternity Hospital in the Area of COVID-19 Reported (The Effect of COVID-19 Pandemic and Social Restrictions on Depression Rates and Maternal Attachment in Immediate Postpartum Women: a Preliminary Study) | Unmet DARE definition of SR |
| Pastick[42] | A Systematic Review of Treatment and Outcomes of Pregnant Women With COVID-19-A Call for Clinical Trials | Unmet DARE definition of SR |
| Priputnevich[43] | The novel coronavirus SARS-COV-2 and pregnancy: Literature review | Unmet DARE definition of SR |
| Rafat[44] | Setting realistic goals for feeding infants when their mothers have suspected or confirmed COVID-19 | Unmet DARE definition of SR |
| Ramadhani[45] | Can remdesivir treat COVID-19 effectively in hospitalized pregnancies?: A literature review | Unmet DARE definition of SR |
| Rodrigues[46] | Pregnancy and breastfeeding during COVID-19 pandemic: A systematic review of published pregnancy cases | Unmet DARE definition of SR |
| Rolnik[47] | Can COVIDâ€19 in pregnancy cause preâ€eclampsia? | Unmet DARE definition of SR |
| Samantha[48] | Psychological impact of infectious disease outbreaks on pregnant women: Rapid evidence review | Wrong population |
| Sampieri[49] | [Review of new evidence about the possible vertical transmission of coronavirus disease-2019] | Unmet DARE definition of SR |
| Shorey[50] | Lessons from past epidemics and pandemics and a way forward for pregnant women, midwives and nurses during COVID-19 and beyond: A meta-synthesis | Wrong population |
| Silva[51] | Is SARS-CoV-2 Vertically Transmitted? | Unmet DARE definition of SR |
| Silva[52] | Immunological aspects of coronavirus disease during pregnancy: an integrative review | Unmet DARE definition of SR |
| Stumpfe[53] | SARS-CoV-2 Infection in Pregnancy - a Review of the Current Literature and Possible Impact on Maternal and Neonatal Outcome | Unmet DARE definition of SR |
| Tobaiqy[54] | Therapeutic Management of COVID-19 Patients: A systematic review | Wrong population |
| Torre[55] | [Recommendations and practical management of pregnant women with COVID-19: a scoping review] | Unmet DARE definition of SR |
| Vieira[56] | Repercussions of the covid-19 pandemic on the mental health of pregnant and puerperal women: a systematic review | Wrong population |
| Walker[57] | Maternal transmission of SARS-COV-2 to the neonate, and possible routes for such transmission: A systematic review and critical analysis | Unmet DARE definition of SR |
| Wingert[58] | Risk factors for severe outcomes of COVID-19: a rapid review | Wrong population |
| Yaqian[59] | Clinical and pathological characteristics of 2019 novel coronavirus disease (COVID-19): a systematic review | Unmet DARE definition of SR |
| Yeo[60] | Review of guidelines and recommendations from 17 countries highlights the challenges that clinicians face caring for neonates born to mothers with COVID-19 | Unmet DARE definition of SR |

**References**

1. Ashary N, Bhide A, Chakraborty P, Colaco S, Mishra A, Chhabria K, et al. Single-Cell RNA-seq Identifies Cell Subsets in Human Placenta That Highly Expresses Factors Driving Pathogenesis of SARS-CoV-2. Frontiers in Cell and Developmental Biology. 2020;8. doi: 10.3389/fcell.2020.00783.

2. Azarkish F, Janghorban R. Impact of COVID-19 infection on maternal and neonatal outcomes: a review of 287 pregnancies. medRxiv. 2020:2020.05.09.20096842. doi: 10.1101/2020.05.09.20096842.

3. Boyadzhieva Vladimira V, Stoilov Nikolay R, Stoilov Rumen M. Coronavirus disease 2019 (COVID-19) during pregnancy in patients with rheumatic diseases. Rheumatology international. 2020.

4. Brown S. Perinatal mental health and the COVID ‐19 pandemic. World Psychiatry. 2020;(3 vo 19).

5. Cabero-Pérez MJ, Gómez-Acebo I, Dierssen-Sotos T, Llorca J. [Infection by SARS-CoV-2 in pregnancy and possibility of transmission to neonates: A systematic revision]. Semergen. 2020;46 Suppl 1:40-7. doi: 10.1016/j.semerg.2020.06.011. PubMed PMID: 32646729.

6. Campos LS, Peixoto Caldas J, M. . Increasing maternal mortality associated with COVID‐19 and shortage of intensive care is a serious concern in low‐resource settings. Acta Obstetricia et Gynecologica Scandinavica. 2020;(10 vo 99).

7. Capobianco G, Saderi L, Aliberti S, Mondoni M, Piana A, Dessole F, et al. COVID-19 in pregnant women: A systematic review and meta-analysis. European Journal of Obstetrics and Gynecology and Reproductive Biology. 2020;252:543-58. doi: 10.1016/j.ejogrb.2020.07.006.

8. Chamseddine RS, Wahbeh F, Chervenak F, Salomon LJ, Ahmed B, Rafii A. Pregnancy and Neonatal Outcomes in SARS-CoV-2 Infection: a systematic review. medRxiv. 2020:2020.05.11.20098368. doi: 10.1101/2020.05.11.20098368.

9. Chang TH, Wu JL, Chang LY. Clinical characteristics and diagnostic challenges of pediatric COVID-19: A systematic review and meta-analysis. Journal of the Formosan Medical Association. 2020;119(5):982-9. doi: 10.1016/j.jfma.2020.04.007.

10. Dawei L, Lin S, Shihua D, Tao L, Yange C, Xiu‐An Y. Asymptomatic COVID‐19 infection in late pregnancy indicated no vertical transmission. Journal of Medical Virology. 2020;(preaccepted).

11. Delfino M, Guida M, Patrì A, Spirito L, Gallo L, Fabbrocini G. SARS‐CoV‐2 possible contamination of genital area: implications for sexual and vertical transmission routes. Journal of the European Academy of Dermatology and Venereology. 2020;(8 vo 34).

12. Delgado A, Santos Lira LC, Soligo Takemoto ML, Katz L, Amorim MM. Rates of Maternal and Perinatal Mortality and Vertical Transmission in Pregnancies Complicated by Severe Acute Respiratory Syndrome Coronavirus 2 (SARS-Co-V-2) Infection: A Systematic Review. Obstetrics and gynecology. 2020;136(4):849. doi: 10.1097/AOG.0000000000004111.

13. Di Mascio D, Khalil A, Saccone G, Rizzo G, Buca D, Liberati M, et al. Outcome of coronavirus spectrum infections (SARS, MERS, COVID-19) during pregnancy: a systematic review and meta-analysis. American Journal of Obstetrics and Gynecology MFM. 2020;2(2). doi: 10.1016/j.ajogmf.2020.100107.

14. Di Nardo M, van Leeuwen G, Loreti A, Barbieri MA, Guner Y, Locatelli F, et al. A literature review of 2019 novel coronavirus (SARS-CoV2) infection in neonates and children. Pediatric Research. 2020. doi: 10.1038/s41390-020-1065-5.

15. Dubey P, Reddy SY, Manuel S, Dwivedi AK. Maternal and neonatal characteristics and outcomes among COVID-19 infected women: An updated systematic review and meta-analysis. European Journal of Obstetrics and Gynecology and Reproductive Biology. 2020;252:490-501. doi: 10.1016/j.ejogrb.2020.07.034.

16. Duran P, Berman S, Niermeyer S, Jaenisch T, Forster T, de Leon RGP, et al. COVID-19 and newborn health: systematic review. Rev Panam Salud Publica. 2020;44:14. doi: 10.26633/rpsp.2020.54. PubMed PMID: WOS:000529448000001.

17. Elshafeey F, Magdi R, Hindi N, Elshebiny M, Farrag N, Mahdy S, et al. A systematic scoping review of COVID-19 during pregnancy and childbirth. International journal of gynaecology and obstetrics: the official organ of the International Federation of Gynaecology and Obstetrics. 2020;150(1):47-52. doi: 10.1002/ijgo.13182. PubMed PMID: 32330287.

18. Furlan MCR, Jurado SR, Uliana CH, da Silva MEP, Negata LA, Maia ACF. A Systematic Review of Pregnancy and Coronavirus Infection: Maternal, Fetal and Neonatal Outcomes. Rev Cuid. 2020;11(2):15. doi: 10.15649/cuidarte.1211. PubMed PMID: WOS:000576632600013.

19. Galang RR, Chang K, Strid P, Snead MC, Woodworth KR, House LD, et al. Severe Coronavirus Infections in Pregnancy: A Systematic Review. Obstetrics and gynecology. 2020;136(2):262-72. doi: 10.1097/AOG.0000000000004011.

20. Gillian AR, Nikhil CP, Fionnuala MM, Moshe H, Chittaranjan NP. Clinical update on COVID ‐19 in pregnancy: A review article. Journal of Obstetrics and Gynaecology Research. 2020;(8 vo 46).

21. Hoekstra E, Neumann K, Boot P, Paassen J, Arbous S. Corticosteroid use in COVID-19 patients: A systematic review and meta-analysis on clinical outcomes. ResearchSquare. 2020. doi: 10.21203/rs.3.rs-52240/v1.

22. Ibrahim E, Ibrahim NE. Review of published systematic reviews and meta-analyses on COVID-19. medRxiv. 2020:2020.06.03.20121137. doi: 10.1101/2020.06.03.20121137.

23. IETSI. Instituto de Evaluación de Tecnologías en Salud e Investigación. SARS-COV-2 mother-fetus transmission: rapid synthesis of evidence. Lima, Perú.2020.

24. Irani M, Pakfetrat A, Mask Mahin K. Novel coronavirus disease 2019 and perinatal outcomes. Journal of education and health promotion. 2020.

25. Jahangir M, Nawaz M, Nanjiani D, Siddiqui MS. Clinical manifestations and outcomes of COVID-19 in the paediatric population: a systematic review. Hong Kong Med J. 2020. doi: 10.12809/hkmj208646. PubMed PMID: 32994372.

26. Juan J, Gil MM, Rong Z, Zhang Y, Yang H, Poon LCY. Effects of Coronavirus Disease 2019 (COVID-19) on Maternal, Perinatal and Neonatal Outcomes: a Systematic Review of 266 Pregnancies. medRxiv. 2020:2020.05.02.20088484. doi: 10.1101/2020.05.02.20088484.

27. Juan J. COVID-19/SARS-CoV-2 News from Preprints; Effects of Coronavirus Disease 2019 (COVID-19) on Maternal, Perinatal and Neonatal Outcomes: a Systematic Review of 266 Pregnancies. Medical Letter on the CDC & FDA. 2020.

28. Kahathuduwa CN, Dhanasekara CS, Chin S-H. Case fatality rate in COVID-19: a systematic review and meta-analysis. Cold Spring Harbor Laboratory; 2020.

29. Khalil A. Coronavirus - COVID-19; Findings from A. Khalil and Co-Researchers Advance Knowledge in COVID-19 Outcome of Coronavirus spectrum infections (SARS, MERS, COVID 1 -19) during pregnancy: a systematic review and meta-analysis. Medical Letter on the CDC & FDA. 2020.

30. Khalili MA, Leisegang K, Majzoub A, Finelli R, Panner Selvam MK, Henkel R, et al. Male Fertility and the COVID-19 Pandemic: Systematic Review of the Literature. The world journal of men's health. 2020. doi: 10.5534/wjmh.200134. PubMed PMID: 32814369.

31. Klaritsch P, Ciresa-Konig A, Pristauz-Telsnigg G, Oeggg. COVID-19 During Pregnancy and Puerperium - A Review by the Austrian Society of Gynaecology and Obstetrics (OEGGG). Geburtshilfe Frauenheilkd. 2020;80(08):813-9. doi: 10.1055/a-1207-0702. PubMed PMID: WOS:000560717800029.

32. Lubbe W, Botha E, Niela-Vilen H, Reimers P. Breastfeeding during the COVID-19 pandemic - a literature review for clinical practice. International breastfeeding journal. 2020;15(1):82. doi: 10.1186/s13006-020-00319-3. PubMed PMID: 32928250.

33. Mark EG, Golden WC, Gilmore MM, Sick-Samuels A, Curless MS, Nogee LM, et al. Community-Onset SARS-CoV-2 Infection in Young Infants: A Systematic Review. The Journal of pediatrics. 2020. doi: 10.1016/j.jpeds.2020.09.008. PubMed PMID: 32910943.

34. Martins-Filho PR, Santo VS, Santos Junior HP. [To breastfeed or not to breastfeed? Lack of evidence on the presence of SARS-CoV-2 in breastmilk of pregnant women with COVID-19]. Rev panam salud pública. 2020;44.

35. Mascarenhas VHA, Caroci-Becker A, Venâncio K, Baraldi NG, Durkin AC, Riesco MLG. COVID-19 and the production of knowledge regarding recommendations during pregnancy: a scoping review. Revista latino-americana de enfermagem. 2020;28:e3348. doi: 10.1590/1518-8345.4523.3348. PubMed PMID: 32609284.

36. Melekhina ЕV, Gorelov AV, Muzyka AD. Clinical characteristics of covid-19 in children of different ages. Literature review as of april 2020. Voprosy Prakticheskoi Pediatrii. 2020;15(2):7-20. doi: 10.20953/1817-7646-2020-2-7-20.

37. Mojgan K-Z, Hossein N, Seyed Alireza D, Hajar A, Seyed Reza M, Athena B, et al. Vertical Transmission of Coronavirus Disease 19 (COVID-19) from Infected Pregnant Mothers to Neonates: A Review. Fetal and Pediatric Pathology. 2020;(3 vo 39).

38. Mullins E, Evans D, Viner RM, Brien PO, Morris E. Coronavirus in pregnancy and delivery: rapid review. Ultrasound in Obstetrics & Gynecology. 2020;(5 vo 55).

39. Mullis E. COVID-19/SARS-CoV-2 News from Preprints; Coronavirus in Pregnancy and Delivery: Rapid Review and Expert Consensus (Published March 8, 2020). Medical Letter on the CDC & FDA. 2020.

40. Mullins E. Coronavirus - COVID-19; Recent Findings from North Bristol NHS Trust Has Provided New Data on COVID-19 (Coronavirus in pregnancy and delivery: rapid review). Medical Letter on the CDC & FDA. 2020.

41. Oskovi-Kaplan ZA, Buyuk GN, Ozgu-Erdinc AS, Keskin HL, Ozbas A, Moraloglu Tekin O. The Effect of COVID-19 Pandemic and Social Restrictions on Depression Rates and Maternal Attachment in Immediate Postpartum Women: a Preliminary Study. Psychiatric Quarterly. 2020. doi: 10.1007/s11126-020-09843-1.

42. Pastick KA, Nicol MR, Smyth E, Zash R, Boulware DR, Rajasingham R, et al. A Systematic Review of Treatment and Outcomes of Pregnant Women With COVID-19-A Call for Clinical Trials. Open forum infectious diseases. 2020;7(9):ofaa350. doi: 10.1093/ofid/ofaa350. PubMed PMID: 32929403.

43. Priputnevich TV, Gordeev AB, Lyubasovskaya LA, Shabanova NE. The novel coronavirus SARS-COV-2 and pregnancy: Literature review. Akusherstvo i Ginekologiya (Russian Federation). 2020;2020(5):6-12. doi: 10.18565/aig.2020.5.6-12.

44. Rafat M, Bosco P. Setting realistic goals for feeding infants when their mothers have suspected or confirmed COVID‐19. Acta Paediatrica. 2020;(10 vo 109).

45. Ramadhani P, Sumarno. Can remdesivir treat COVID-19 effectively in hospitalized pregnancies?: A literature review. Journal of Pharmaceutical Sciences and Research. 2020;12(7):992-1000.

46. Rodrigues C, Baia I, Domingues R, Barros H. Pregnancy and breastfeeding during COVID-19 pandemic: A systematic review of published pregnancy cases. medRxiv. 2020:2020.04.25.20079509. doi: 10.1101/2020.04.25.20079509.

47. Rolnik DL. Can COVID‐19 in pregnancy cause pre‐eclampsia? BJOG: An International Journal of Obstetrics & Gynaecology. 2020;(11 vo 127).

48. Samantha KB, Dale W, Neil G. Psychological impact of infectious disease outbreaks on pregnant women: Rapid evidence review. medRxiv. 2020. doi: 10.1101/2020.04.16.20068031.

49. Sampieri CL, Montero H. [Review of new evidence about the possible vertical transmission of coronavirus disease-2019]. Gaceta sanitaria. 2020. doi: 10.1016/j.gaceta.2020.06.005. PubMed PMID: 32711871.

50. Shorey S, Chan V. Lessons from past epidemics and pandemics and a way forward for pregnant women, midwives and nurses during COVID-19 and beyond: A meta-synthesis. Midwifery. 2020;90:102821. doi: 10.1016/j.midw.2020.102821.

51. Silva A, Leal CRV. Is SARS-CoV-2 Vertically Transmitted? Front Pediatr. 2020;8:5. doi: 10.3389/fped.2020.00276. PubMed PMID: WOS:000541114300001.

52. Silva C, Oliveira LV, Lopes LP, Santos W, Agra IKR. Immunological aspects of coronavirus disease during pregnancy: an integrative review. Revista da Associacao Medica Brasileira (1992). 2020;66(5):696-700. doi: 10.1590/1806-9282.66.5.696. PubMed PMID: 32638966.

53. Stumpfe FM, Titzmann A, Schneider MO, Stelzl P, Kehl S, Fasching PA, et al. SARS-CoV-2 Infection in Pregnancy - a Review of the Current Literature and Possible Impact on Maternal and Neonatal Outcome. Geburtshilfe und Frauenheilkunde. 2020;80(4):380-90. doi: 10.1055/a-1134-5951.

54. Tobaiqy M, Qashqary M, Al-Dahery S, Mujallad A, Hershan AA, Kamal MA, et al. Therapeutic Management of COVID-19 Patients: A systematic review. Cold Spring Harbor Laboratory; 2020.

55. Torre HG, Rodríguez-Rodríguez R, Martínez AM. [Recommendations and practical management of pregnant women with COVID-19: a scoping review]. Enfermeria clinica. 2020. doi: 10.1016/j.enfcli.2020.05.009. PubMed PMID: 32425489.

56. Vieira LG, SIlva Camargo EL, Schneider G, Pereira Rocatti da Silva G, Thomazini M, Possani MA, et al. Repercussions of the covid-19 pandemic on the mental health of pregnant and puerperal women: a systematic review. medRxiv. 2020. doi: 10.1101/2020.08.17.20176560.

57. Walker KF, O’Donoghue K, Grace N, Dorling J, Comeau JL, Li W, et al. Maternal transmission of SARS-COV-2 to the neonate, and possible routes for such transmission: A systematic review and critical analysis. BJOG: An International Journal of Obstetrics and Gynaecology. 2020. doi: 10.1111/1471-0528.16362.

58. Wingert A, Pillay J, Gates M, Guitard S, Rahman S, Beck A, et al. Risk factors for severe outcomes of COVID-19: a rapid review. Cold Spring Harbor Laboratory; 2020.

59. Yaqian m, Lin W, Wen J, Chen G. Clinical and pathological characteristics of 2019 novel coronavirus disease (COVID-19): a systematic review. Cold Spring Harbor Laboratory; 2020.

60. Yeo KT, Oei JL, De Luca D, Schmolzer GM, Guaran R, Palasanthiran P, et al. Review of guidelines and recommendations from 17 countries highlights the challenges that clinicians face caring for neonates born to mothers with COVID-19. Acta Paediatr. 2020:16. doi: 10.1111/apa.15495. PubMed PMID: WOS:000560744700001.
