# Supplementary material for: COVID-19 and pregnancy: An umbrella review of clinical presentation, vertical transmission, and maternal and perinatal outcomes
Source: PLoS One. 2021 Jun 29;16(6):e0253974. doi: 10.1371/journal.pone.0253974 (PMC8241118; doi:10.1371/journal.pone.0253974)
Supplement: S7 File — (DOCX) [file pone.0253974.s007.docx]

# S7 File.1 Quality assessment of systematic reviews by the 16 AMSTAR-2 items

| **Authors (2020)** | 1* | 2* | 3* | 4* | 5* | 6* | 7* | 8* | 9 RCT* | 9 NRS* | 10* | 11 RCT* | 11 NRS* | 12* | 13* | 14* | 15* | 16* | **# Non-negative** | **Overall confidence** |
| --- | --- | --- | --- | --- | --- | --- | --- | --- | --- | --- | --- | --- | --- | --- | --- | --- | --- | --- | --- | --- |
| **AbdelMassih[**[**1**](#_ENREF_1)**]** | Yes | N | N | PY | N | Y | N | N | N | N | N | N MA | N MA | N MA | N | N | N | Y | 6 | Critically low |
| **Abdollahpour[**[**2**](#_ENREF_2)**]** | Y | PY | N | Y | Y | Y | N | Y | NA | N | N | N MA | N MA | N MA | N | N | N MA | Y | 10 | Critically low |
| **Akhtar[**[**3**](#_ENREF_3)**]** | Y | N | Y | Y | Y | Y | N | Y | NA | Y | N | N MA | N MA | N MA | N | Y | N MA | Y | 12 | Critically low |
| **Allotey[**[**4**](#_ENREF_4)**]** | Y | Y | Y | Y | Y | Y | N | Y | NA | Y | N | NA | Y | Y | Y | Y | Y | Y | 14 | Low |
| **Arabi[**[**5**](#_ENREF_5)**]** | Y | N | Y | PY | Y | N | N | Y | NA | N | N | Y | Y | Y | Y | N | N | N | 8 | Critically low |
| **Ashraf[**[**6**](#_ENREF_6)**]** | Y | PY | Y | Y | N | N | N | Y | NA | N | N | N MA | N MA | N MA | N | N | N MA | Y | 9 | Critically low |
| **Banaei[**[**7**](#_ENREF_7)**]** | Y | N | Y | Y | Y | Y | N | Y | NA | Y | N | N MA | N MA | N MA | N | N | N MA | Y | 11 | Critically low |
| **Bwire[**[**8**](#_ENREF_8)**]** | Y | Y | Y | Y | Y | Y | N | Y | NA | N | N | N MA | N MA | N MA | Y | N | N MA | Y | 12 | Critically low |
| **Caparros Gonzalez[**[**9**](#_ENREF_9)**]** | Y | N | Y | Y | N | N | N | N | NA | N | N | N MA | N MA | N MA | N | N | N MA | N | 6 | Critically low |
| **Centeno-Tablante[**[**10**](#_ENREF_10)**]** | Y | Y | Y | Y | Y | Y | N | Y | NA | PY | N | NA | N MA | N MA | Y | Y | N MA | Y | 14 | Critically low |
| **Chi[**[**11**](#_ENREF_11)**]** | Y | PY | Y | PY | Y | N | N | Y | NA | N | N | N MA | N MA | N MA | N | N | N MA | Y | 10 | Critically low |
| **de Sousa[**[**12**](#_ENREF_12)**]** | Y | N | Y | Y | N | N | N | Y | NA | N | N | N MA | N MA | N MA | N | N | N MA | Y | 8 | Critically low |
| **Della Gatta[**[**13**](#_ENREF_13)**]** | Y | Y | Y | PY | Y | Y | N | Y | NA | Y | N | N MA | N MA | N MA | N | N | N MA | N | 11 | Critically low |
| **Deniz[**[**14**](#_ENREF_14)**]** | Y | N | Y | PY | N | N | N | Y | NA | N | N | N MA | N MA | N MA | N | N | N MA | N | 7 | Critically low |
| **Dhir[**[**15**](#_ENREF_15)**]** | Y | N | N | PY | Y | Y | N | Y | NA | N | N | N MA | N | N MA | N | N | N | N | 6 | Critically low |
| **Di Mascio[**[**16**](#_ENREF_16)**]** | Y | Y | Y | PY | Y | Y | N | Y | NA | N | N | N MA | Y | N | N | Y | N | Y | 10 | Critically low |
| **Diriba[**[**17**](#_ENREF_17)**]** | Y | N | Y | PY | Y | Y | N | Y | NA | Y | N | N MA | Y | N | Y | Y | Y | Y | 12 | Critically low |
| **Duran[**[**18**](#_ENREF_18)**]** | Y | N | N | PY | N | N | N | Y | NA | N | N | N MA | N MA | N MA | N | N | N MA | Y | 7 | Critically low |
| **Figueiro-Filho[**[**19**](#_ENREF_19)**]** | Y | N | N | Y | Y | Y | N | Y | NA | N | N | N MA | N MA | N MA | N | N | N MA | Y | 9 | Critically low |
| **Furlan[**[**20**](#_ENREF_20)**]** | Y | N | Y | Y | Y | Y | N | Y | NA | N | N | N MA | N MA | N MA | N | N | N MA | Y | 10 | Critically low |
| **Gajbhiye[**[**21**](#_ENREF_21)**]** | Y | N | N | PY | Y | N | N | Y | NA | N | N | N MA | N MA | N MA | N | N | N MA | Y | 8 | Critically low |
| **Gao[**[**22**](#_ENREF_22)**]** | Y | N | N | Y | Y | Y | N | Y | NA | N | N | Y | Y | N | N | Y | Y | Y | 9 | Critically low |
| **Goh[**[**23**](#_ENREF_23)**]** | Y | N | N | PY | N | N | N | N | NA | N | N | N | N | N | N | N | N | Y | 3 | Critically low |
| **Gordon[**[**24**](#_ENREF_24)**]** | Y | PY | Y | PY | Y | Y | N | Y | NA | Y | N | N MA | N MA | N MA | Y | N | N MA | Y | 13 | Critically low |
| **Han[**[**25**](#_ENREF_25)**]** | Y | N | N | PY | N | N | N | PY | NA | N | N | NA | Y | N | N | N | N | Y | 7 | Critically low |
| **Hasan[**[**26**](#_ENREF_26)**]** | Y | PY | N | Y | Y | N | N | Y | NA | N | N | N MA | N MA | N MA | Y | Y | N MA | Y | 11 | Critically low |
| **Hessami[**[**27**](#_ENREF_27)**]** | Y | N | N | PY | N | N | N | Y | NA | N | N | N MA | N MA | N MA | N | N | N MA | Y | 7 | Critically low |
| **Huntley[**[**28**](#_ENREF_28)**]** | Y | PY | Y | Y | Y | Y | N | Y | NA | Y | N | N MA | N MA | N MA | Y | Y | N MA | Y | 14 | Low |
| **Juan[**[**29**](#_ENREF_29)**]** | Y | Y | N | Y | Y | Y | Y | Y | NA | Y | N | N MA | N MA | N MA | Y | Y | N MA | Y | 14 | Moderate |
| **Kasraeian[**[**30**](#_ENREF_30)**]** | Y | N | N | Y | N | N | N | Y | N | N | N | Y | Y | N | N | N | Y | Y | 6 | Critically low |
| **Khalil[**[**31**](#_ENREF_31)**]** | Y | Y | N | PY | Y | Y | Y | Y | NA | PY | N | NA | Y | Y | N | Y | Y | Y | 13 | Critically low |
| **Khan[**[**32**](#_ENREF_32)**]** | Y | N | Y | N | N | Y | N | Y | NA | Y | N | N MA | N MA | N MA | N | N | N MA | Y | 9 | Critically low |
| **Kotlyar[**[**33**](#_ENREF_33)**]** | Y | Y | N | Y | Y | N | N | Y | NA | PY | N | N | N | N | N | N | N | Y | 7 | Critically low |
| **Li[**[**34**](#_ENREF_34)**]** | Y | PY | Y | Y | Y | Y | Y | PY | NA | PY | N | N MA | N MA | N MA | N | N | N MA | N | 7 | Critically low |
| **Martins[**[**35**](#_ENREF_35)**]** | Y | N | N | N | N | N | N | Y | NA | N | N | N MA | N MA | N MA | N | N | N MA | N | 5 | Critically low |
| **Matar[**[**36**](#_ENREF_36)**]** | Y | N | N | Y | N | N | N | Y | NA | Y | N | N MA | Y | N | N | Y | Y | N | 7 | Critically low |
| **Melo[**[**37**](#_ENREF_37)**]** | Y | N | Y | PY | Y | Y | N | Y | NA | Y | N | N MA | Y | N | N | Y | N | N | 9 | Critically low |
| **Authors (2020)** | 1* | 2* | 3* | 4* | 5* | 6* | 7* | 8* | 9 RCT* | 9 NRS* | 10* | 11 RCT* | 11 NRS* | 12* | 13* | 14* | 15* | 16* | **# Non-negative** | **Overall confidence** |
| **Mirbeyk[**[**38**](#_ENREF_38)**]** | Y | N | Y | Y | N | N | N | Y | NA | N | N | N MA | N MA | N MA | N | N | N MA | Y | 8 | Critically low |
| **Muhidin[**[**39**](#_ENREF_39)**]** | Y | N | Y | Y | Y | Y | N | Y | NA | Y | N | N MA | N MA | N MA | N | N | N MA | Y | 11 | Critically low |
| **Mullins[**[**40**](#_ENREF_40)**]** | Y | N | Y | N | N | N | N | Y | NA | N | N | N MA | N MA | N MA | N | N | N MA | Y | 7 | Critically low |
| **Mustafa[**[**41**](#_ENREF_41)**]** | Y | N | N | PY | N | N | N | N | NA | N | N | N MA | N MA | N MA | N | N | N MA | Y | 6 | Critically low |
| **Panahi[**[**42**](#_ENREF_42)**]** | Y | N | Y | Y | Y | N | N | Y | NA | PY | N | N MA | N MA | N MA | N | N | N MA | Y | 10 | Critically low |
| **Pettirosso[**[**43**](#_ENREF_43)**]** | Y | N | Y | PY | N | N | Y | Y | NA | N | N | N MA | N MA | N MA | Y | Y | N MA | Y | 11 | Critically low |
| **Rahman[**[**44**](#_ENREF_44)**]** | Y | N | Y | Y | N | N | N | Y | NA | N | N | N MA | N MA | N MA | N | N | N MA | Y | 8 | Critically low |
| **Raschetti[**[**45**](#_ENREF_45)**]** | Y | PY | Y | Y | Y | Y | N | Y | NA | N | N | N MA | N MA | N MA | N | N | N MA | Y | 11 | Critically low |
| **Rodrí­guez-Blanco[**[**46**](#_ENREF_46)**]** | Y | N | Y | PY | N | Y | N | Y | NA | Y | N | N MA | N MA | N MA | N | N | N MA | Y | 10 | Critically low |
| **Rostami[**[**47**](#_ENREF_47)**]** | Y | N | N | Y | N | N | N | Y | NA | N | N | N MA | N MA | N MA | N | N | N MA | Y | 7 | Critically low |
| **Segars[**[**48**](#_ENREF_48)**]** | Y | N | N | PY | Y | N | N | Y | NA | N | N | N MA | N MA | N MA | Y | N | N MA | N | 8 | Critically low |
| **Shi[**[**49**](#_ENREF_49)**]** | Y | N | N | PY | N | N | N | Y | NA | N | N | N MA | Y | N | N | Y | N | Y | 6 | Critically low |
| **Singh[**[**50**](#_ENREF_50)**]** | Y | N | N | Y | Y | Y | N | Y | NA | N | N | N MA | N MA | N MA | N | N | N MA | Y | 9 | Critically low |
| **Smith[**[**51**](#_ENREF_51)**]** | Y | PY | Y | Y | Y | N | N | Y | NA | Y | N | N MA | N MA | N MA | Y | N | N MA | Y | 12 | Low |
| **Soheili[**[**52**](#_ENREF_52)**]** | Y | N | Y | Y | Y | Y | N | Y | NA | Y | N | N MA | Y | N | N | N | Y | Y | 10 | Critically low |
| **Teles Abrao Trad[**[**53**](#_ENREF_53)**]** | Y | N | N | PY | Y | N | N | Y | NA | N | N | N MA | N MA | N MA | N | N | N MA | Y | 8 | Critically low |
| **Thomas[**[**54**](#_ENREF_54)**]** | Y | N | N | PY | N | N | N | N | NA | Y | N | N MA | N MA | N MA | N | N | N MA | Y | 7 | Critically low |
| **Trevisanuto[**[**55**](#_ENREF_55)**]** | Y | N | Y | Y | Y | Y | N | Y | NA | Y | Y | N MA | N MA | N MA | N | N | N MA | Y | 12 | Critically low |
| **Trippella[**[**56**](#_ENREF_56)**]** | Y | N | Y | Y | Y | Y | Y | Y | NA | Y | N | N MA | N MA | N MA | Y | Y | N MA | Y | 14 | Low |
| **Trocado[**[**57**](#_ENREF_57)**]** | Y | N | Y | PY | Y | Y | N | Y | NA | Y | N | N MA | N MA | N MA | N | N | N MA | Y | 11 | Critically low |
| **Turan[**[**58**](#_ENREF_58)**]** | Y | N | N | PY | Y | N | N | N | NA | N | N | N MA | N MA | N MA | N | N | N MA | Y | 7 | Critically low |
| **Uygun-Can[**[**59**](#_ENREF_59)**]** | Y | N | N | PY | Y | N | N | Y | NA | Y | N | N MA | Y | N | Y | N | Y | N | 8 | Critically low |
| **Vakili[**[**60**](#_ENREF_60)**]** | Y | N | N | PY | N | N | N | Y | NA | N | N | N MA | N MA | N MA | Y | N | N MA | Y | 8 | Critically low |
| **Yang N[**[**61**](#_ENREF_61)**]** | Y | Y | N | PY | Y | Y | N | Y | NA | Y | N | N MA | N MA | N MA | N | N | N MA | Y | 11 | Critically low |
| **Yang Z[**[**62**](#_ENREF_62)**]** | Y | PY | N | PY | Y | Y | N | Y | NA | N | N | N MA | N MA | N MA | N | N | N MA | Y | 10 | Critically low |
| **Yang Z[**[**63**](#_ENREF_63)**]** | Y | N | N | Y | Y | Y | N | Y | NA | N | N | N MA | N MA | N MA | N | N | N MA | Y | 9 | Critically low |
| **Yee[**[**64**](#_ENREF_64)**]** | Y | N | Y | Y | Y | Y | N | Y | NA | N | N | N | N | N | N | N | N | Y | 7 | Critically low |
| **Yoon[**[**65**](#_ENREF_65)**]** | Y | N | N | Y | Y | Y | N | Y | NA | N | N | N MA | N MA | N MA | Y | Y | N MA | Y | 11 | Critically low |
| **Zaigham[**[**66**](#_ENREF_66)**]** | Y | N | N | PY | Y | N | N | N | NA | Y | N | N MA | N MA | N MA | N | N | N MA | Y | 8 | Critically low |

*** The 16 AMSTAR-2 items:**

1. Did the research questions and inclusion criteria for the review include the components of PICO?
2. Did the report of the review contain an explicit statement that the review methods prior to the conduct of the review and did the report justify any significant deviations from the protocol?
3. Did the review authors explain their selection of the study designs for inclusion in the review?
4. Did the review authors use a comprehensive literature search strategy?
5. Did the review authors perform study selection in duplicate?
6. Did the review authors perform data extraction in duplicate?
7. Did the review authors provide a list of excluded studies and justify the exclusions?
8. Did the review authors describe the included studies in adequate detail?
9. Did the review authors use a satisfactory technique for assessing the risk of bias (RoB) in individual studies that were included in the review? (For RCT and NRS)
10. Did the review authors report on the sources of funding for the studies included in the review?
11. If meta-analysis was performed did the review authors use appropriate methods for statistical combination of results? (For RCT and NRS)
12. If meta-analysis was performed, did the review authors assess the potential impact of RoB in individual studies on the results of the meta-analysis or other evidence synthesis?
13. Did the review authors account for RoB in individual studies when interpreting/discussing the results of the review?
14. Did the review authors provide a satisfactory explanation for, and discussion of, any heterogeneity observed in the results of the review?
15. If they performed quantitative synthesis did the review authors carry out an adequate investigation of publication bias (small study bias) and discuss its likely impact on the results of the review?
16. Did the review authors report any potential sources of conflict of interest, including any funding they received for conducting the review?

*If the answers to all signaling questions for a domain are ‘‘yes’’ or ‘‘probably yes,’’ then level of concern can be judged as low. If any signaling question is answered ‘‘no’’ or ‘‘probably no,’’ potential for concern about bias exists.

# S7.2 Quality assessment of systematic reviews by ROBIS

| **Authors (2020)** | **Phase 2: concerns with the review process** | | | | **Phase 3*** |
| --- | --- | --- | --- | --- | --- |
|  | **1. Study eligibility criteria** | **2. Identification and selection of studies** | **3. Data collection and study appraisal** | **4. Synthesis and findings** | **Risk of bias in the review** |
| **Allotey[**[**4**](#_ENREF_4)**]** | **☺️** | **☺️** | **☺️** | **☺️** | **☺️ Low** |
| **Centeno-Tablante[**[**10**](#_ENREF_10)**]** | **☺️** | **☺️** | **☺️** | **?** | **? Unclear** |
| **Figueiro-Filho[**[**19**](#_ENREF_19)**]** | **☺️** | **?** | **☹** | **?** | **☹ High** |
| **Juan[**[**29**](#_ENREF_29)**]** | **☹** | **☺️** | **☺️** | **?** | **☹ High** |

*If the answers to all signaling questions for a domain are ‘‘yes’’ or ‘‘probably yes,’’ then level of concern can be judged as low. If any signaling question is answered ‘‘no’’ or ‘‘probably no,’’ potential for concern about bias exists.

**REFERENCES**

1. AbdelMassih A, Fouda R, Essam R, Negm A, Khalil D, Habib D, et al. COVID-19 during pregnancy should we really worry from vertical transmission or rather from fetal hypoxia and placental insufficiency? A systematic review and meta -analysis. 2020. doi: 10.21203/rs.3.rs-71847/v1.

2. Abdollahpour S, Khadivzadeh T. Improving the quality of care in pregnancy and childbirth with coronavirus (COVID-19): a systematic review. J Matern-Fetal Neonatal Med. 2020:9. doi: 10.1080/14767058.2020.1759540. PubMed PMID: WOS:000534953600001.

3. Akhtar H, Patel C, Abuelgasim E, Harky A. COVID-19 (SARS-CoV-2) Infection in Pregnancy: A Systematic Review. Gynecologic and Obstetric Investigation. 2020. doi: 10.1159/000509290.

4. Allotey J, Stallings E, Bonet M, Yap M, Chatterjee S, Kew T, et al. Clinical manifestations, risk factors, and maternal and perinatal outcomes of coronavirus disease 2019 in pregnancy: living systematic review and meta-analysis. BMJ (Clinical research ed). 2020;370:m3320. doi: 10.1136/bmj.m3320.

5. Arabi S, Vaseghi G, Heidari Z, Shariati L, Amin B, Rashid H, et al. Clinical characteristics of COVID-19 infection in pregnant women: a systematic review and meta-analysis. medRxiv. 2020:2020.04.05.20053983. doi: 10.1101/2020.04.05.20053983.

6. Ashraf MA, Keshavarz P, Hosseinpour P, Erfani A, Roshanshad A, Pourdast A, et al. Coronavirus disease 2019 (COVID-19): A systematic review of pregnancy and the possibility of vertical transmission. Journal of Reproduction and Infertility. 2020;21(3):157-68.

7. Banaei M, Ghasemi V, Saei M, Naz MSG, Kiani Z, Rashidi-Fakari F, et al. Obstetrics and Neonatal Outcomes in Pregnant Women with COVID-19: A Systematic Review. Iran J Public Health. 2020;49:38-47. PubMed PMID: WOS:000531776100006.

8. Bwire GM, Njiro BJ, Mwakawanga DL, Sabas D, Sunguya BF. Possible vertical transmission and antibodies against SARS-CoV-2 among infants born to mothers with COVID-19: A living systematic review. J Med Virol. 2020. doi: 10.1002/jmv.26622. PubMed PMID: 33090535.

9. CaparrosGonzalez Rafael A. Maternal and neonatal consequences of coronavirus COVID-19 infection during pregnancy: a scoping review. Revista espanola de salud publica. 2020.

10. Centeno-Tablante E, Medina-Rivera M, Finkelstein JL, Rayco-Solon P, Garcia-Casal MN, Rogers L, et al. Transmission of SARS-CoV-2 through breast milk and breastfeeding: a living systematic review. Annals of the New York Academy of Sciences. 2020. doi: 10.1111/nyas.14477.

11. Chi H, Chiu NC, Tai YL, Chang HY, Lin CH, Sung YH, et al. Clinical features of neonates born to mothers with coronavirus disease-2019: A systematic review of 105 neonates. Journal of microbiology, immunology, and infection = Wei mian yu gan ran za zhi. 2020. doi: 10.1016/j.jmii.2020.07.024. PubMed PMID: 32847748.

12. de Sousa AFL, de Carvalho HEF, de Oliveira LB, Schneider G, Camargo ELS, Watanabe E, et al. Effects of COVID-19 Infection during Pregnancy and Neonatal Prognosis: What Is the Evidence? Int J Environ Res Public Health. 2020;17(11):17. doi: 10.3390/ijerph17114176. PubMed PMID: WOS:000542629600429.

13. Della Gatta AN, Rizzo R, Pilu G, Simonazzi G. Coronavirus disease 2019 during pregnancy: a systematic review of reported cases. American Journal of Obstetrics and Gynecology. 2020;223(1):36-41. doi: 10.1016/j.ajog.2020.04.013.

14. Deniz M, Tezer H. Vertical transmission of SARS CoV-2: a systematic review. Journal of Maternal-Fetal and Neonatal Medicine. 2020:1-8. doi: 10.1080/14767058.2020.1793322.

15. Dhir SK, Kumar J, Meena J, Kumar P. Clinical Features and Outcome of SARS-CoV-2 Infection in Neonates: A Systematic Review. Journal of tropical pediatrics. 2020. doi: 10.1093/tropej/fmaa059.

16. Di Mascio D, Khalil A, Saccone G, Rizzo G, Buca D, Liberati M, et al. Outcome of Coronavirus spectrum infections (SARS, MERS, COVID 1 -19) during pregnancy: a systematic review and meta-analysis. American Journal of Obstetrics & Gynecology MFM. 2020;2(2):100107. doi: 10.1016/j.ajogmf.2020.100107.

17. Diriba K, Awulachew E, Getu E. The effect of coronavirus infection (SARS-CoV-2, MERS-CoV, and SARS-CoV) during pregnancy and the possibility of vertical maternal-fetal transmission: a systematic review and meta-analysis. European journal of medical research. 2020;25(1):39. doi: 10.1186/s40001-020-00439-w.

18. Duran P, Berman S, Niermeyer S, Jaenisch T, Forster T, Ponce de Leon RG, et al. COVID-19 and newborn health: systematic review^ien. Rev panam salud pública. 2020;44.

19. Figueiro-Filho EA, Yudin M, Farine D. COVID-19 during pregnancy: an overview of maternal characteristics, clinical symptoms, maternal and neonatal outcomes of 10,996 cases described in 15 countries. J Perinat Med. 2020. doi: 10.1515/jpm-2020-0364. PubMed PMID: 33001856.

20. Furlan MCR, Jurado SR, Uliana CH, Silva MEP, Nagata LA, Maia ACF. Gravidez e infecção por Coronavírus: desfechos maternos, fetais e neonatais ­ Revisão sistemática^iptA Systematic Review of Pregnancy and Coronavirus Infection: Maternal, Fetal and Neonatal Outcomes^ienRevisión sistemática del embarazo y la infección por. rev cuid (Bucaramanga 2010). 2020;11(2).

21. Gajbhiye R, Modi D, Mahale S. Pregnancy outcomes, Newborn complications and Maternal-Fetal Transmission of SARS-CoV-2 in women with COVID-19: A systematic review of 441 cases. medRxiv. 2020:2020.04.11.20062356. doi: 10.1101/2020.04.11.20062356.

22. Gao YJ, Ye L, Zhang JS, Yin YX, Liu M, Yu HB, et al. Clinical features and outcomes of pregnant women with COVID-19: A systematic review and meta-analysis. BMC Infectious Diseases. 2020;20(1). doi: 10.1186/s12879-020-05274-2.

23. Goh XL, Low YF, Ng CH, Amin Z, Ng YPM. Incidence of SARS-CoV-2 vertical transmission: a meta-analysis. Archives of disease in childhood Fetal and neonatal edition. 2020. doi: 10.1136/archdischild-2020-319791.

24. Gordon M, Kagalwala T, Rezk K, Rawlingson C, Ahmed MI, Guleri A. Rapid systematic review of neonatal COVID-19 including a case of presumed vertical transmission. BMJ Paediatrics Open. 2020;4(1). doi: 10.1136/bmjpo-2020-000718.

25. Han Y, Ma H, Suo M, Han F, Wang F, Ji J, et al. Clinical manifestation, outcomes in pregnant women with COVID-19 and the possibility of vertical transmission: a systematic review of the current data. J Perinat Med. 2020. doi: 10.1515/jpm-2020-0431. PubMed PMID: 33068387.

26. Hasan MZ, Kibria GMA, Alam T. Pregnancy during the evolving pandemic Coronavirus Disease 2019 (COVID-19): A rapid scoping review of early evidence in the published literature. Research Square; 2020.

27. Hessami K, Homayoon N, Hashemi A, Vafaei H, Kasraeian M, Asadi N. COVID-19 and maternal, fetal and neonatal mortality: a systematic review. Journal of Maternal-Fetal and Neonatal Medicine. 2020. doi: 10.1080/14767058.2020.1806817.

28. Huntley BJF, Huntley ES, Di Mascio D, Chen T, Berghella V, Chauhan SP. Rates of Maternal and Perinatal Mortality and Vertical Transmission in Pregnancies Complicated by Severe Acute Respiratory Syndrome Coronavirus 2 (SARS-Co-V-2) Infection: A Systematic Review. Obstetrics and gynecology. 2020;136(2):303-12. doi: 10.1097/AOG.0000000000004010.

29. Juan J, Gil MM, Rong Z, Zhang Y, Yang H, Poon LC. Effect of coronavirus disease 2019 (COVID-19) on maternal, perinatal and neonatal outcome: systematic review. Ultrasound in obstetrics & gynecology : the official journal of the International Society of Ultrasound in Obstetrics and Gynecology. 2020;56(1):15-27. doi: 10.1002/uog.22088.

30. Kasraeian M, Zare M, Vafaei H, Asadi N, Faraji A, Bazrafshan K, et al. COVID-19 pneumonia and pregnancy; a systematic review and meta-analysis. Journal of Maternal-Fetal and Neonatal Medicine. 2020. doi: 10.1080/14767058.2020.1763952.

31. Khalil A, Kalafat E, Benlioglu C, O'Brien P, Morris E, Draycott T, et al. SARS-CoV-2 infection in pregnancy: A systematic review and meta-analysis of clinical features and pregnancy outcomes. EClinicalMedicine. 2020;25. doi: 10.1016/j.eclinm.2020.100446.

32. Khan MMA, Khan MN, Mustagir MG, Rana J, Haque MR, Rahman MM. COVID-19 infection during pregnancy: A systematic review to summarize possible symptoms, treatments, and pregnancy outcomes. Cold Spring Harbor Laboratory; 2020.

33. Kotlyar AM, Grechukhina O, Chen A, Popkhadze S, Grimshaw A, Tal O, et al. Vertical transmission of coronavirus disease 2019: a systematic review and meta-analysis. American Journal of Obstetrics and Gynecology. 2020. doi: 10.1016/j.ajog.2020.07.049.

34. Li W, Tang J, Zeng Y, Yue Y, He Y, Zhang M, et al. A systematic review of SARS-infected pregnant females, newborns, children and adolescents. Chinese Journal Of Evidence-Based Medicine. 2020;20(04):426-36.

35. Martins PR, Santos VS, Santos HP. To breastfeed or not to breastfeed? Lack of evidence on the presence of SARS-CoV-2 in breastmilk of pregnant women with COVID-19. Rev Panam Salud Publica. 2020;44:7. doi: 10.26633/rpsp.2020.59. PubMed PMID: WOS:000529448200001.

36. Matar R, Alrahmani L, Monzer N, Debiane LG, Berbari E, Fares J, et al. Clinical Presentation and Outcomes of Pregnant Women with COVID-19: A Systematic Review and Meta-Analysis. Clinical infectious diseases : an official publication of the Infectious Diseases Society of America. 2020. doi: 10.1093/cid/ciaa828.

37. Melo GC, Araújo K. COVID-19 infection in pregnant women, preterm delivery, birth weight, and vertical transmission: a systematic review and meta-analysis. Cadernos de saude publica. 2020;36(7):e00087320. doi: 10.1590/0102-311x00087320. PubMed PMID: 32696830.

38. Mirbeyk M, Rezaei N. The impact of COVID-19 on pregnancy and neonatal health: a systematic review. Research Square; 2020.

39. Muhidin S, Behboodi Moghadam Z, Vizheh M. Analysis of Maternal Coronavirus Infections and Neonates Born to Mothers with 2019-nCoV; a Systematic Review. Archives of academic emergency medicine. 2020;8(1):e49. doi: 10.22037/AAEM.V8I1.656.G788.

40. Mullins E, Evans D, Viner R, O'Brien P, Morris E. Coronavirus in pregnancy and delivery: rapid review and expert consensus. Cold Spring Harbor Laboratory; 2020.

41. Mustafa NM, L AS. Characterisation of COVID-19 Pandemic in Paediatric Age Group: A Systematic Review and Meta-Analysis. Journal of clinical virology : the official publication of the Pan American Society for Clinical Virology. 2020;128:104395. doi: 10.1016/j.jcv.2020.104395. PubMed PMID: 32417675.

42. Panahi L, Amiri M, Pouy S. Clinical Characteristics of COVID-19 Infection in Newborns and Pediatrics: A Systematic Review. Archives of academic emergency medicine. 2020;8(1):e50. PubMed PMID: 32440661.

43. Pettirosso E, Giles M, Cole S, Rees M. COVID-19 and pregnancy: A review of clinical characteristics, obstetric outcomes and vertical transmission. Australian and New Zealand Journal of Obstetrics and Gynaecology. 2020. doi: 10.1111/ajo.13204.

44. Rahman HS, Aziz MS, Hussein RH, Othman HH, Salih Omer SH, Khalid ES, et al. The transmission modes and sources of COVID-19: A systematic review. International Journal of Surgery Open. 2020;26:125-36. doi: 10.1016/j.ijso.2020.08.017.

45. Raschetti R, Vivanti AJ, Vauloup-Fellous C, Loi B, Benachi A, De Luca D. Synthesis and systematic review of reported neonatal SARS-CoV-2 infections. Nature Communications. 2020;11(1). doi: 10.1038/s41467-020-18982-9.

46. Rodríguez-Blanco N, Vegara-Lopez I, Aleo-Giner L, Tuells J. [Scoping review of coronavirus case series (SARS-CoV, MERS-CoV and SARS-CoV-2) and their obstetric and neonatal results]. Revista espanola de quimioterapia : publicacion oficial de la Sociedad Espanola de Quimioterapia. 2020. doi: 10.37201/req/064.2020. PubMed PMID: 32683837.

47. Rostami M, Mansouritorghabeh H. D-dimer level in COVID-19 infection: a systematic review. Expert Rev Hematol. 2020. doi: 10.1080/17474086.2020.1831383. PubMed PMID: 32997543.

48. Segars J, Katler Q, McQueen DB, Kotlyar A, Glenn T, Knight Z, et al. Prior and novel coronaviruses, Coronavirus Disease 2019 (COVID-19), and human reproduction: what is known? Fertility and sterility. 2020;113(6):1140-9. doi: 10.1016/j.fertnstert.2020.04.025. PubMed PMID: 32482250.

49. Shi L, Wang Y, Yang H, Duan G. Laboratory Abnormalities in Pregnant Women with Novel Coronavirus Disease 2019. American journal of perinatology. 2020;37(10):1070-3. doi: 10.1055/s-0040-1712181. PubMed PMID: 32396949.

50. Singh B, Gornet M, Sims H, Kisanga E, Knight Z, Segars J. Severe Acute Respiratory Syndrome-Corona Virus-2 (SARS-CoV-2) and its Effect on Gametogenesis and Early Pregnancy. Am J Reprod Immunol. 2020:e13351. doi: 10.1111/aji.13351. PubMed PMID: 32969123.

51. Smith V, Seo D, Warty R, Payne O, Salih M, Chin KL, et al. Maternal and neonatal outcomes associated with COVID-19 infection: A systematic review. PLoS ONE. 2020;15(6). doi: 10.1371/journal.pone.0234187.

52. Soheili M, Moradi G, Baradaran HR, Soheili M, Moradi Y. Clinical Manifestation and Maternal Complications and Neonatal outcomes in Pregnant Women with COVID 19: An Update a Systematic Review and Meta-analysis. Research Square; 2020.

53. Teles Abrao Trad A, Ibirogba ER, Elrefaei A, Narang K, Tonni G, Picone O, et al. Complications and outcomes of SARS-CoV-2 in pregnancy: where and what is the evidence? Hypertension in pregnancy. 2020;39(3):361-9. doi: 10.1080/10641955.2020.1769645. PubMed PMID: 32456489.

54. Thomas P, Alexander PE, Ahmed U, Elderhorst E, El-Khechen H, Mammen MJ, et al. Vertical transmission risk of SARS-CoV-2 infection in the third trimester: a systematic scoping review. Journal of Maternal-Fetal and Neonatal Medicine. 2020. doi: 10.1080/14767058.2020.1786055.

55. Trevisanuto D, Cavallin F, Cavicchiolo ME, Borellini M, Calgaro S, Baraldi E. Coronavirus infection in neonates: A systematic review. Archives of Disease in Childhood: Fetal and Neonatal Edition. 2020. doi: 10.1136/archdischild-2020-319837.

56. Trippella G, Ciarcia M, Ferrari M, Buzzatti C, Maccora I, Azzari C, et al. COVID-19 in Pregnant Women and Neonates: A Systematic Review of the Literature with Quality Assessment of the Studies. Pathogens. 2020;9(6):25. doi: 10.3390/pathogens9060485. PubMed PMID: WOS:000551563600001.

57. Trocado V, Silvestre-Machado J, Azevedo L, Miranda A, Nogueira-Silva C. Pregnancy and COVID-19: a systematic review of maternal, obstetric and neonatal outcomes. Journal of Maternal-Fetal and Neonatal Medicine. 2020:1-13. doi: 10.1080/14767058.2020.1781809.

58. Turan O, Hakim A, Dashraath P, Jeslyn WJL, Wright A, Abdul-Kadir R. Clinical characteristics, prognostic factors, and maternal and neonatal outcomes of SARS-CoV-2 infection among hospitalized pregnant women: A systematic review. International Journal of Gynecology and Obstetrics. 2020;151(1):7-16. doi: 10.1002/ijgo.13329.

59. Uygun-Can B, Acar-Bolat B. Clinical Properties and Diagnostic Methods of COVID-19 Infection in Pregnancies: Meta-Analysis. BioMed Research International. 2020:1-8. doi: 10.1155/2020/1708267. PubMed PMID: 146144524. Language: English. Entry Date: In Process. Revision Date: 20201001. Publication Type: Article. Journal Subset: Biomedical.

60. Vakili S, Savardashtaki A, Jamalnia S, Tabrizi R, Nematollahi MH, Jafarinia M, et al. Laboratory Findings of COVID-19 Infection are Conflicting in Different Age Groups and Pregnant Women: A Literature Review. Archives of Medical Research. 2020. doi: 10.1016/j.arcmed.2020.06.007.

61. Yang N, Che S, Zhang J, Wang X, Tang Y, Wang J, et al. Breastfeeding of infants born to mothers with COVID-19: A rapid review. Annals of Translational Medicine. 2020;8(10). doi: 10.21037/atm-20-3299.

62. Yang Z, Liu Y. Vertical Transmission of Severe Acute Respiratory Syndrome Coronavirus 2: A Systematic Review. American Journal of Perinatology. 2020;37(1):1055-60. doi: 10.1055/s-0040-1712161.

63. Yang Z, Wang M, Zhu Z, Liu Y. Coronavirus disease 2019 (COVID-19) and pregnancy: a systematic review. Journal of Maternal-Fetal and Neonatal Medicine. 2020. doi: 10.1080/14767058.2020.1759541.

64. Yee J, Kim W, Han JM, Yoon HY, Lee N, Lee KE, et al. Clinical manifestations and perinatal outcomes of pregnant women with COVID-19: a systematic review and meta-analysis. Sci Rep. 2020;10(1):18126. doi: 10.1038/s41598-020-75096-4. PubMed PMID: 33093582.

65. Yoon SH, Kang JM, Ahn JG. Clinical outcomes of 201 neonates born to mothers with COVID-19: A systematic review. European Review for Medical and Pharmacological Sciences. 2020;24(14):7804-15. doi: 10.26355/eurrev_202007_22285.

66. Zaigham M, Andersson O. Maternal and perinatal outcomes with COVID-19: A systematic review of 108 pregnancies. Acta Obstetricia et Gynecologica Scandinavica. 2020;99(7):823-9. doi: 10.1111/aogs.13867.
