# Supplementary material for: COVID-19 and pregnancy: An umbrella review of clinical presentation, vertical transmission, and maternal and perinatal outcomes
Source: PLoS One. 2021 Jun 29;16(6):e0253974. doi: 10.1371/journal.pone.0253974 (PMC8241118; doi:10.1371/journal.pone.0253974)
Supplement: S8 File — (DOCX) [file pone.0253974.s008.docx]

# S8 File. Systematic review level numerical data by research question

## ***Table 1: Clinical presentation in SARS-COV2 pregnant women***

| Authors (2020) | N pregnant women | Asympto-matic | Headache | Myalgia | Fatigue/  malaise | Fever | Diarrhea | Mild respiratory symptoms | Dyspnea/  Shortness of breath | Pneumonia | Sepsis | Mechanical  ventilation/  IUC | Death |
| --- | --- | --- | --- | --- | --- | --- | --- | --- | --- | --- | --- | --- | --- |
| **AbdelMassih[**[**1**](#_ENREF_1)**]** | 1787 | 219/1787 (12%) | 60/1787 (3.3%) | 56/1787 (3%) | NA | 951/1787 (53%) | 78/1787 (4.4%) | 934/1787 (52.2%) | 417/1787 (23.3%) | NA | NA | 130/1787 (7%) | NA |
| **Akhtar[**[**2**](#_ENREF_2)**]** | 156 | NA | NA | 17/156 (11%) | 13% | 82/156 (53%) | NA | 50/156 (32%) | 12/156 (8%) | NA | NA | 17/156 (11%) | NA |
| **Allotey[**[**3**](#_ENREF_3)**]** | depends on each outcome | 115/2380  (4.8%) | NA | 1411/6078  (23.21%) | NA | 2733/8328  (32.81%) | 659/7525  (8.74%) | 3432/8317 (41.26%) | 1928/8159(23.63%) | 729/2577  (28.88%) | 7/737 (0.94%) | 155/10713  (1.44%) | 73/11 580 (0.63%) |
| **Arabi[**[**4**](#_ENREF_4)**]** | 50 | NA | NA | 5/50 (1%) | 0/50 (0%) | 38/50 (77%) | 0/50 (0%) | 10/50 (20%) | 5/50 (10%) | NA | NA | NA | 0/50 (0%) |
| **Ashraf[**[**5**](#_ENREF_5)**]** | 90 | NA | 3/90 (3.3%) | 6/90 (6.6%) | 11/90  (12.2%) | 47/90 (52.2%) | 1/90 (1.1%) | 34/90 (37.7%) | 12/90  (13.3%) | 59/90 (65%) | 1/90 (1.1%) | 3/90 (3.3%) | 1/90 (1.1%) |
| **de Sousa[**[**6**](#_ENREF_6)**]** | 755 | 83/689 (12%) | NA | 51/689 (7.4%) | 69/689 (10%) | 363/689 (52.7%) | 29/689 (4.2%) | NA | 78/689 (11.4%) | NA | NA | NA | NA |
| **Della Gatta[**[**7**](#_ENREF_7)**]** | 51 | 13 (31%) | NA | 3/35 (8.6%) | 3/35 (8.6%) | 17/35 (48%) | 2/35 (5.7%) | 16/35 (46%) | 4/35 (11.4%) | NA | NA | NA | NA |
| **Diriba[**[**8**](#_ENREF_8)**]** | depends on each outcome | NA | 88/674  (13.05%) | 61/293  (20.8%) | 192/634  (20.34%) | 575/870 (66%) | NA | 458/869  (52.7%) | 49/303  (16%) | 368/517(71.2%) | NA | NA | 15/557  (2.69%) |
| **Figueiro-Filho[**[**9**](#_ENREF_9)**]** | depends on each outcome | 814/6598 (6.2%) | 1409/3474 (40.69%) | 1861/4290 (43.49%) | 55/680 (8.1%) | 2050/5138 (40%) | 597/5154 (11.6%) | 2667/5154 (51.75%) | 1303/5154 (51.75%) | NA | NA | 170/1243 (13.7%) | NA |
| **Furlan[**[**10**](#_ENREF_10)**]** | 399 | NA | NA | 8/19 (42.1%) | NA | 87/157 (55.4%) | NA | 46/119 (38.6%) | 7/32  (21.8%) | NA | NA | 6/35 (17.1%) | 2/284 (0.7%) |
| **Gajbhiye[**[**11**](#_ENREF_11)**]** | 441 | NA | NA | 70/369 (18.9) | NA | 206/369 (55.8) | NA | 159/369( 43%) | NA | 30/369 (8.1%) | NA | 31/369 (8.4%) | 4/369 (1%) |
| **Gao[**[**12**](#_ENREF_12)**]** | 236 | NA | NA | NA | NA | 120/236 (51%) | NA | 73/236 (31%) | NA | NA | NA | NA | NA |
| **Han[**[**13**](#_ENREF_13)**]** | depends on each outcome | NA | NA | 83/678 (12.68%) | NA | 568/954 (59.54%) | 37/787 (4.7%) | 506/952 (53.15%) | 241/885  (27.23%) | NA | NA | 36/221  (16.28%) | 13/1005(1.29%) |
| **Huntley[**[**14**](#_ENREF_14)**]** | 538 | NA | 15/161 (9.3%) | 26/156 (16.7%) | 52/337 (15.4%) | 238/496 (48%) | NA | 211/463 (45.6%) | 274/364 (75.2%) | NA | NA | 8/263 | 0/348 (0%) |
| **Juan[**[**15**](#_ENREF_15)**]** | 324 | NA | NA | 30/315(9.5%) | 29/315 (9%) | 154/315 (48.8%) | 14/315 (4.4%) | 117/315 (37%) | 46/315 (14.6%) | 190/198 (90.5%) | NA | 12/188 | 9/315 (285) |
| **Kasraeian[**[**16**](#_ENREF_16)**]** | 87 | 28/87 (30%) | NA | NA | NA | 37/87 (86%) | NA | 50/87 (68%) | NA | 87/87 (100%) | NA | NA | 0/87 (0%) |
| **Khalil[**[**17**](#_ENREF_17)**]** | 2567 | 253/1205 (14.5%) | 92/640 (15.0%) | 104/640 (18.9%) | 101/545 (18.5%) | 1292/1987 (63.3%) | 126/1621 (7.4%) | 391/1987 (71.4%) | 789/1941 (34.4%) | NA | NA | 92/1680 (3.4%) | 43/2468 (0.9%) |
| **Khan[**[**18**](#_ENREF_18)**]** | 101 | NA | 3/101 (3%) | 7/101 (7.1%) | 15/101 (15.2%) | 66/101 (66.7%) | 4/101 (4%) | 39/101 (39.4%) | 14/101 (14.1%) | NA | NA | NA | NA |
| **Li[**[**19**](#_ENREF_19)**]** | 19 | NA | NA | NA | NA | 19/19 (100%) | NA | 15/19 (78.94%) | NA | NA | NA | 5/19 (26.31%) | 3/19 (15.78%) |
| **Matar[**[**20**](#_ENREF_20)**]** | 136 | NA | NA | NA | NA | 75/136 (63%) | 21/136 (15.6%) | 39/130 (37%) | 21/136 (15.7%) | NA | NA | NA | 1/136 (11.1%) |
| **Mirbeyk[**[**21**](#_ENREF_21)**]** | 364 | 25/364 (7%) | NA | NA | NA | 227/364 (62.4%) | NA | 165/364 (45.3%) | NA | 22/364 (6%) | NA | NA | 2/364 (0.55%) |
| **Mullins[**[**22**](#_ENREF_22)**]** | 19 | 3/19 (16%) | NA | NA | NA | NA | NA | NA | NA | NA | NA | 1/19 (5%) | NA |
| **Pettirosso[**[**23**](#_ENREF_23)**]*** | 3830 | 178/510 (34.9%) | 89/198 (45%) | 137/350 (39%) | 72/317 (22.7%) | 458/989 (46.3%) | 15/295 (5%) | NA | 117/474 (24.6%) | NA | NA | NA | NA |
| **Rahman[**[**24**](#_ENREF_24)**]** | 102 | NA | NA | 15/64 (23,4%) | NA | 55/64 (85.9%) | 7/79  (8.9%) | 26/79 (32.9%) | 11/79 (13.9%) | 53/70 (75.7%) | NA | 3/70 (4,3%) | 0/79  (0%) |
| **Smith[**[**25**](#_ENREF_25)**]** | 92 | 30/92 (32.6%) | NA | NA | 25/82 (30.49%) | 57/92 (61.9%) | NA | 35/92 (38.4%) | 10/83 (12.1%) | 78/79 (98.7%) | NA | 1/92 (1.1%) | 2/92 (2.2%) |
| **Soheili[**[**26**](#_ENREF_26)**]** | 177 | NA | NA | 10/58 (18%) | 8/58 (13%) | 71/126 (56%) | 6/65 (9%) | 33/110 (30%) | 5/176 (3%) | NA | NA | NA | NA |
| **Teles Abrao Trad[**[**27**](#_ENREF_27)**]** | 139 | 29/139 (20%) | NA | NA | NA | 80/139 (57.6%) | NA | 44/139 (31.7%) | 19/139 (13.7%) | NA | NA | 5/155  (3.2%) | NA |
| **Thomas[**[**28**](#_ENREF_28)**]** | 157 | 26/63 (41%) | NA | 7/93  (7.52%) | 6/93  (6.45%) | 67/93  (72%) | 6/93  (6.45%) | 27/93  (29%) | 6/93 (6.45%) | NA | NA | NA | 1/93 (1%) |
| **Trippella[**[**29**](#_ENREF_29)**]** | 275 | 22/269 (8%) | NA | 37/269  (13.8%) | 28/275 (10.2%) | 155/275  (56.3%) | 9/275  (3.27%) | 98/275  (35.6%) | 9/275  (3.3%) | 162/275 (59%) | NA | NA | 1/275  (0.36%) |
| **Trocado[**[**30**](#_ENREF_30)**]** | 95 | NA | NA | 6/95 (6%) | 10/95 (11%) | 52/95 (55%) | 4/95 (4%) | 36/95 (38%) | 7/95(8%) | NA | NA | NA | NA |
| **Turan[**[**31**](#_ENREF_31)**]** | 637 | 68/637 (10.7%) | NA | 79/515 (15.3%) | NA | 273/515 (53%) | 18/515 (3.5%) | 224/515 (43.5%) | 64/515 (12.4%) | NA | NA | 51/61 (83.6%) | 1.6% (10/637) |
| **Uygun-Can[**[**32**](#_ENREF_32)**]** | 181 | NA | NA | NA | NA | 69/181 (38%) | NA | 40/181 (22%) | 6/181 (3.3%). | NA | NA | NA | NA |
| **Yang Z[**[**33**](#_ENREF_33)**]** | 114 | NA | NA | 18/114 (16.3%) | 26/114 (22.5%) | 100/114 (87.5%) | 10/114  (8.8%) | 61/114 (53.8%) | 13/114 (11.3%) | NA | NA | NA | NA |
| **Yee[**[**34**](#_ENREF_34)**]** | 9032 | NA | NA | 1354/8372 (16.2%) | 60/127 (54.5%) | 1395/8571 (27.6%) | 505/8310 (6.5%) | 2018/8560 (50.1%) | 1130/8560 (20.7%) | NA | NA | NA | NA |
| **Yoon[**[**35**](#_ENREF_35)**]** | 223 | NA | NA | 12/56  (21.4%) | NA | 85/201 (42.4%) | NA | 64/201  (31.8%) | 16/142 (42.4%) | NA | NA | NA | NA |
| **Zaigham[**[**36**](#_ENREF_36)**]** | 108 | NA | NA | 11/108  (10.2%) | 14/108  (12.9%) | 63/92 (68%) | 7/108  (6.5%) | 37/108 (40%) | 13/108 (14%) | NA | NA | NA | 0/108 (0%) |

*Information calculated by the authors

## ***Table 2: Laboratory findings in SARS-COV2 pregnant women***

| Author | N pregnant women | Increased reactive c protein | Lymphocytopenia | Leukocytosis  / neutrophilia | Trombocyto-penia | Elevated ALT or AST | Elevated d-dimer | Radiology pneumonia | Ground-glass opacities in CT | Infiltrated shadows in CT | Signs of pneumonia XR or CT |
| --- | --- | --- | --- | --- | --- | --- | --- | --- | --- | --- | --- |
| **Allotey[**[**3**](#_ENREF_3)**]** | depends on each outcome | 174/426  (40.84%) | 262/780(33.59%) | 50/251  (19.92%) | 36/428  (8.41%) | 51/491  (10.39%) | NA | NA | 246/387  (63.56%) | NA | 599/1968  (30.43%) |
| **Ashraf[**[**5**](#_ENREF_5)**]** | 90 | 26/32 (81.2%) | 18/28 (64.3%) | 11/24 (45.8%) | 44/46 (95.7%) | 8/25 (32%) | NA | 59/60 (99%) | 59/60 (99%) | 59/60 (99%) | 59/60 (99%) |
| **deSousa[**[**6**](#_ENREF_6)**]** | 755 | 260/475 (54.7%) | 257/475 (54.1%) | Leukocytosis 168/475 (35.4%)  Neutrophilia 40/475 (8.4%) | NA | (ALT) 39/475 (8.2%)  (AST) 45/475 (9.5%) | NA | NA | NA | NA | NA |
| **Diriba[**[**8**](#_ENREF_8)**]** | depends on each outcome | 80/143  (55.94%) | 92/146  (63%) | 27/95  (28%) | NA | 9/48  (18.75%) | NA | NA | 50/76  (66.6%) | 11/19  (57.89%) | NA |
| **Figueiro-Filho[**[**9**](#_ENREF_9)**]** | depends on each outcome | 162/592 (27.59%) | 146/182 (80.2%) | NA | NA | 48/427 (27.49%) | 86/385 (22.39%) | NA | NA | NA | NA |
| **Furlan[**[**10**](#_ENREF_10)**]** | 399 | NA | NA | NA | 6/35  (17.14%) | 46/119 (38.6%) | 87/157  (55.4%) | 7/32  (21.8%) | NA | NA | 8/19 (42.1%) |
| **Gao[**[**12**](#_ENREF_12)**]** | 236 | NA | 116/236 (49%) | NA | NA | NA | NA | NA | NA | NA | 168/236 (7.1%) |
| **Han[**[**13**](#_ENREF_13)**]** | depends on each outcome | NA | 137/383 (35.8%) | NA | NA | 14/71 (19.7%) | NA | NA | NA | NA | 420/866 (48.5%) |
| **Huntley[**[**14**](#_ENREF_14)**]** | 538 | NA | 110/232 (47.4%) | NA | NA | NA | NA | NA | NA | NA | NA |
| **Juan[**[**15**](#_ENREF_15)**]** | 324 | 104/213  (48.8%) | 99/213(46%) | NA | NA | 12/54(22%) | NA | NA | NA | NA | 190/198(96%) |
| **Kasraeian[**[**16**](#_ENREF_16)**]** | 87 | NA | 24/87 (70%) | NA | NA | NA | NA | NA | NA | NA | NA |
| **Khalil[**[**17**](#_ENREF_17)**]** | 2567 | 144/351 (54.0%;) | 143/444 (34.2%) | NA | 7/259 | 48/318 (16.0%) | 77/91  (2.5%) | NA | NA | NA | NA |
| **Li[**[**19**](#_ENREF_19)**]** | 19 | NA | 7/18 (38.8%) | NA | 7/18 (38.8%) | NA | NA | NA | NA | NA | 19/19 (100%) |
| **Matar[**[**20**](#_ENREF_20)**]** | 136 | 43/76 (57%) | 36/72; (50%) | NA | NA | (ALT) 10/44 (22.3%)  (AST) 11/51 (23.3%) | NA | NA | 71/87 (81.7%) | 5/11 (42.5%) | NA |
| **Muhidin[**[**22**](#_ENREF_22)**]** | 89 | 29/30 (96%) | 19 /30  (63%) | NA | NA | 3/30 (10%) | NA | NA | NA | NA | NA |
| **Shi[**[**37**](#_ENREF_37)**]** | 173 | 104/151 (69%) | 97/164 (59%) | 37/46 (81%) | NA | NA | 89/109 (82%) | NA | NA | NA | NA |
| **Smith[**[**25**](#_ENREF_25)**]** | 92 | NA | 66.7% (46/69) | NA | NA | NA | NA | NA | NA | NA | NA |
| **Soheili[**[**26**](#_ENREF_26)**]** | 177 | 30/51 (58%) | 46/126 (37%) | NA | NA | NA | NA | NA | NA | NA | NA |
| **TelesAbraoTrad[**[**27**](#_ENREF_27)**]** | 139 | NA | 32/90 (35.6%) | 8/90 (8.8%) | NA | NA | NA | NA | NA | NA | 82/155 (53.5% ) |
| **Trippella[**[**29**](#_ENREF_29)**]** | 275 | 52/108 (48.1%) | 31/108  (7%) | NA | NA | 9/108  (26.85%) | NA | 48/48 | NA | 162/171  (95%) | NA |
| **Turan[**[**31**](#_ENREF_31)**]** | 637 | 275  (72.2%) | 179  (47.0%) | 53 (13.9%) | NA | NA | 94  (24.7%) | NA | NA | NA | 196 (88.7%) |
| **Uygun-Can[**[**32**](#_ENREF_32)**]** | 181 | NA | NA | NA | NA | NA | NA | NA | NA | 177/181 (97.9%) | NA |
| **Vakili[**[**38**](#_ENREF_38)**]** | 54 | 36/54 (66%) | 30/54 (55%) | 41/54 (77%) | NA | 18/54 (33%) | NA | NA | NA | NA | NA |
| **Yee[**[**34**](#_ENREF_34)**]** | 9032 | 86/180 (47.7%) | 108/258 (42.6%) | NA | NA | NA | NA | NA | NA | NA | NA |
| **Yoon[**[**35**](#_ENREF_35)**]** | 223 | 65/103  (63.1%) | 52/120  (43.3%) | 28/89  (31.5%) | NA | NA | NA | NA | NA | NA | 185/200  (92.5%) |
| **Zaigham[**[**36**](#_ENREF_36)**]** | 108 | 45/64  (70%) | 40/68  (58.8%) | NA | NA | NA | NA | NA | NA | NA | NA |

## ***Table 3: Maternal outcomes in SARS-COV2 pregnant women***

| Authors (2020) | N pregnant women | Vaginal delivery | C-section | Gestational diabetes | Preeclampsia | Hypertension | Stillbirth | PROM | Placenta previa | Miscarriage or abortion | Placenta abruptio | Intrauterine growth retardation. | Fetal distress |
| --- | --- | --- | --- | --- | --- | --- | --- | --- | --- | --- | --- | --- | --- |
| AbdelMassih[[1](#_ENREF_1)] | 1787 | NA | NA | NA | NA | NA | NA | NA | NA | NA | NA | NA | 3/71 (7%) |
| Abdollahpour[[39](#_ENREF_39)] | NA | NA | NA | NA | NA | NA | NA | NA | NA | NA | NA | NA | NA |
| Akhtar[[2](#_ENREF_2)] | 156 | 19/108 (1.7%) | 66/108 (61.1%) | NA | NA | NA | NA | 86/108 (8%) | NA | NA | NA | NA | 1/108 (14%) |
| Allotey[[3](#_ENREF_3)] | 13118 | NA | 184/491 (37.5%) | NA | NA | NA | 3/427 (0.7%) | NA | NA | NA | NA | NA | 25/293 (8.5%) |
| Arabi[[4](#_ENREF_4)] | 50 | NA | 30/50 (61%) | NA | 0/50 (0%) | NA | NA | NA | NA | NA | NA | NA | 1/50 (0.1%) |
| Ashraf[[5](#_ENREF_5)] | 90 | 9/90 (10%) | 81/90 (90%) | 4/90 (4.4%) | 2/90 (2.22%) | 2/90 (2.22%) | 1/90 (1.1%) | 6/90 (6.7%) | NA | NA | NA | NA | 15/86 (17,4%) |
| Banaei[[40](#_ENREF_40)] | 123 | 17/123 (13.8%) | 99/123 (80.55) | NA | NA | NA | 1/123 (0.8%) | NA | NA | NA | NA | NA | NA |
| Chi[[41](#_ENREF_41)] | NA | NA | NA | NA | NA | NA | NA | NA | NA | NA | NA | 10/105 (0.9%) | NA |
| Della Gatta[[7](#_ENREF_7)] | 51 | 2/48 (4.2%) | 46/48 (95.8%) | NA | 1/34 (2.9%) | 1/48 (2.1%) | 1/34 (2.9%) | 9/34 (26.5%) | 1/34 (2.9%) | NA | 1/34 (2.9%) | NA | NA |
| Deniz[[42](#_ENREF_42)] | 714 | 105/714 (14%) | 609/714 (85.2%) | NA | NA | NA | NA | 44/714 (6.1%) | NA | NA | NA | NA | NA |
| Dhir[[43](#_ENREF_43)] | 1125 | NA | 742/1125 (66%) | NA | NA | NA | NA | NA | NA | NA | NA | NA | NA |
| Di Mascio[[44](#_ENREF_44)] | 41 | NA | 38/41 (91%) | NA | 1/10 (10%) | NA | 1/42 (2.4%) | 5/31 (18.8%) | NA | NA | NA | NA | 12 /30 (40%) |
| Diriba[[8](#_ENREF_8)] | 1316 | 198/692 (28.6%) | 440/772 (57%) | NA | 10/169 (0.6%) | NA | NA | 17/183 (9.3%) | NA | 9/62 (14.5%) | NA | 3/108 (2.7%) | 14/56 (25%) |
| Figueiro-Filho[[9](#_ENREF_9)] | depends on each outcome | 349/1119 (31.2%) | 761/1119 (68%) | NA | NA | NA | NA | NA | NA | NA | NA | NA | NA |
| Furlan[[10](#_ENREF_10)] | 399 | NA | NA | NA | NA | NA | 1/188 (0.5%) | NA | NA | 1/188 (0.5%) | NA | NA | NA |
| Gajbhiye[[11](#_ENREF_11)] | 441 | NA | NA | 33/369 (8.9%) | 37/369 (10%) | NA | 4/369 (1.1%) | 33/369 (8.9%) | NA | NA | NA | NA | 31/369 (8%) |
| Gao[[12](#_ENREF_12)] | 236 | NA | 153/236 ( 65%) | NA | NA | NA | NA | NA | NA | NA | NA | NA | 68/236 (29%) |
| Han[[13](#_ENREF_13)] | depends on each outcome | 283/733 (38.6%) | 135/586 (23%) | NA | 24/381(6.3%) | NA | 8/663 (1.2%) | 32/176 (18.1%) | NA | 20/743 (2.7%) | NA | NA | NA |
| Huntley[[14](#_ENREF_14)] | 538 | NA | 332/392 (85%) | NA | NA | NA | 0/274 (0.0%) | NA | NA | NA | NA | NA | NA |
| Juan[[15](#_ENREF_15)] | 324 | 51/313 (16,3%) | 186/313 (59.4%) | 20/233 (8.6%) | 6/193 (3.1%) | 14/194 (7.2%) | 4/313 (1.3%) | NA | 1/42(2.38%) | 4/313 (1.3%) | NA | NA | NA |
| Kasraeian[[16](#_ENREF_16)] | 87 | 1/69 (1%) | 64/69 (92%) | NA | NA | NA | 2/87 (0,2%) | 4/31(13.9%) | NA | NA | NA | NA | 10/31 (0,3%) |
| Khalil[[17](#_ENREF_17)] | 2567 | NA | 390/746 (48.3%) | NA | NA | NA | 12/1362 (0.9%) | NA | NA | NA | NA | NA | (15/238 (5.3%) |
| Khan[[18](#_ENREF_18)] | 101 | NA | 50/60 (83.9%) | NA | NA | NA | 0/60 (0%) | NA | NA | NA | NA | NA | NA |
| Li[[19](#_ENREF_19)] | 19 | NA | 9/12(75%) | NA | NA | NA | NA | NA | NA | 4/10(4%) | NA | 3/13 (23%) | NA |
| Martins[[45](#_ENREF_45)] | 24 | NA | 22/24 (91.7%) | NA | NA | NA | NA | NA | NA | NA | NA | NA | NA |
| Matar[[20](#_ENREF_20)] | 136 | NA | 72/ 94 (76.3%) | NA | NA | NA | NA | NA | NA | NA | NA | NA | 3/94(3,2%) |
| Melo[[46](#_ENREF_46)] | 520 | NA | 366/520 (70.4%) | NA | NA | NA | NA | NA | NA | NA | NA | NA | NA |
| Mirbeyk[[21](#_ENREF_21)] | 364 | NA | 257/364 (86%) | NA | NA | NA | NA | NA | NA | NA | NA | NA | NA |
| Muhidin[[47](#_ENREF_47)] | 89 | 5/86 (5.8%) | 79/86 (91.8%) | NA | 1/79 (1.3%) | NA | 2/79 (2.5%) | NA | NA | NA | 6/89 (43.2%) | NA | 15/89 (16.8%) |
| Mullins[[22](#_ENREF_22)] | 19 | 2/19 (10.5%) | 17/19 (89%) | NA | NA | NA | NA | 8/19 (42%) | NA | NA | NA | NA | NA |
| Pettirosso[[23](#_ENREF_23)] | 3830 | NA | NA | NA | NA | NA | 1/13(8%) | 17/160 (10.6%) | NA | 5/159 (3.14%) | NA | NA | NA |
| Rodrí­guez-Blanco[[48](#_ENREF_48)] | NA | 8/73 (11,0%) | 65/73 (89,0%) | NA | NA | 6/64 (8.1%) | NA | 9/74 (12,1%) | NA | NA | NA | 0/74 (0,0%) | NA |
| Segars[[49](#_ENREF_49)] | 162 | NA | NA | NA | NA | NA | NA | NA | NA | 2/162 (1.2%) | NA | 9/162 (5.5%) | 8/60 (13,3%) |
| Smith[[25](#_ENREF_25)] | 92 | 3/50 (6%) | 40/50 (80%) | NA | NA | NA | NA | NA | NA | NA | NA | NA | 11 / 18 (61.1%) |
| Soheili[[26](#_ENREF_26)] | 177 | 25/178 (14%) | 152/177 (86%) | NA | 46/177 (26%) | NA | 3/177 (2%) | NA | NA | NA | NA | NA | 10/65 (15%) |
| Teles Abrao Trad[[27](#_ENREF_27)] | 139 | 9/116 (7.8%) | 107/116 (92.2%) | 10/138 (0.7%) | 4/116 (3.4%) | 5/138(3.6%) | NA | 10/116 (8.6%) | NA | NA | NA | NA | 19 / 18 (16.1%) |
| Thomas[[28](#_ENREF_28)] | 157 | 42/157 (26.7%) | 115/157 (73.2%) | 13/44 (29%) | 6/44 (13.6%) | 5/44 (11.4%) | NA | 7/44 (15.9%) | NA | NA | 1/44 (2.3%) | NA | 6/44 (14%) |
| Trippella[[29](#_ENREF_29)] | 275 | 60/275 (21.8%) | 179/275 (65%) | 10/275 (3.6%) | 5/275 (1.8%) | 10/275 (3.6%) | 2/275 (0.7%) | 3/275 (1%) | NA | NA | NA | NA | NA |
| Trocado[[30](#_ENREF_30)] | 95 | 3/50 (6%) | 47/ 50 (94%) | 3/95 (3%) | 1/95 (1%) | 2/95(2%) | NA | 5/95 (5%) | 2/95 (2%) | NA | NA | NA | 7/51 (14%) |
| Turan[[31](#_ENREF_31)] | 637 | 76/ 479 (15.9%) | 403/479 (84.1%) | NA | NA | NA | 7/500 (1.4%) | NA | NA | 7/86 (8.1%) | NA | NA | 28/60 (46.7) |
| Yang Z[[33](#_ENREF_33)] | 114 | 9/114 (22%) | 89/114 (78%) | NA | NA | NA | 1/114 (0,9%) | NA | NA | NA | NA | NA | 8/75 (10.7%) |
| Yee[[34](#_ENREF_34)] | 9032 | NA | NA | NA | NA | NA | NA | (2/63) 2.4% | NA | NA | NA | NA | 10/63 (15,1%) |
| Yoon[[35](#_ENREF_35)] | 223 | NA | NA | NA | NA | NA | 2/201(1%) | 16/126(12.7%) | NA | NA | NA | NA | NA |
| Zaigham[[36](#_ENREF_36)] | 108 | 7/85 (8.3%) | 78/85 (91.7%) | NA | NA | NA | 1/87 (1.1%) | NA | NA | NA | NA | NA | NA |

## ***Table 4: Clinical presentation in neonates born to SARS COV2 mothers***

| Author | N neonates | Asymptomatic | Reparatory distress syndrome | Sepsis | Mild respiratory symptoms | Gastrointestinal symptoms | Fever | Shortness of breath | Radiology pneumonia | SARS Cov2  (RT-PCR) | Elevated SARSCov2 IgM | Elevated SARSCov2 IgG |
| --- | --- | --- | --- | --- | --- | --- | --- | --- | --- | --- | --- | --- |
| AbdelMassih[[1](#_ENREF_1)] | 71 | NA | NA | NA | NA | NA | 2/71 (4%) | NA | NA | NA | NA | NA |
| Akhtar[[2](#_ENREF_2)] | 108 | NA | NA | NA | NA | 4/108 (4%) | 3/108 (3%) | 6/108 (6%) | NA | NA | NA | NA |
| Allotey[[3](#_ENREF_3)] | 2557 | NA | NA | 2/51 (4%) | NA | NA | NA | NA | NA | NA | NA | NA |
| Ashraf[[5](#_ENREF_5)] | 86 | 19/86 (22.1%) | 2/86 (2.32%) | NA | NA | NA | NA | NA | NA | NA | NA | NA |
| Banaei[[40](#_ENREF_40)] | 123 | NA | 1/123 (0.8%) | NA | 1/123 (0.8%) | 2/123 (1.6%) | 3/123 (2.4%) | 1/123 (0.8%) | 4/123 (3.2%) | 5/123 (4%) | NA | NA |
| Caparros Gonzalez[[50](#_ENREF_50)] | 10 | NA | NA | NA | NA | NA | 2/10 (20%) | 6/10 (60%) | NA | NA | NA | NA |
| Chi[[41](#_ENREF_41)] | 105 | NA | NA | NA | NA | NA | NA | NA | NA | 5/91 (5.5%) | 3/105 (2.8%) | 3/105 (2.8%) |
| de Sousa[[6](#_ENREF_6)] | 598 | NA | NA | NA | NA | NA | NA | NA | NA | 9/493 (2%) | 3/493 (0.6%) | 3/493 (0.6%) |
| Dhir[[43](#_ENREF_43)] | 1141 | 13/45 (22.4%) | NA | NA | NA | 5/45 (8.6%) | 9/45 (15.5%) | 4/45 (8.8%) | NA | 39/1105 (3.9%) | NA | NA |
| Duran[[51](#_ENREF_51)] | 222 | NA | 4/222 (8.8%) | NA | 6/222 (2.7%) | NA | 16 /222 (7.2%) | 1/222 (0.4%) | 7/71(9,8%) | NA | NA | NA |
| Figueiro-Filho [[9](#_ENREF_9)] | Depends of each outcome | NA | 28/576 (4.9%) | 1/241 (0.4%) | NA | NA | NA | NA | NA | 18/1116 (1.6%) | NA | NA |
| Gajbhiye [[11](#_ENREF_11)] | 391 | NA | 31 /369 (8%) | NA | NA | NA | NA | NA | 30 /369 (8%) | 17/313 (5%) | 4/8 (50%) | 7/8 (87,5%) |
| Gordon[[52](#_ENREF_52)] | 10 | NA | NA | NA | NA | 1/10 (1%) | NA | NA | NA | 10/10 (100%) | NA | NA |
| Juan[[15](#_ENREF_15)] | 221 | NA | NA | NA | 2 /79 (2.5%) | NA | 0 /37 (0%) | 0 /37 (0%) | 0 /37 (0%) | NA | NA | NA |
| Mirbeyk[[21](#_ENREF_21)] | 302 | NA | NA | NA | NA | NA | NA | NA | NA | 13 /252 (5%) | NA | NA |
| Muhidin[[47](#_ENREF_47)] | 89 | NA | NA | NA | NA | 5/89 (5,6%) | 3/89 (3,4%) | 6/89 (6,7%) | NA | 0/89 (0%) | NA | NA |
| Mullins[[22](#_ENREF_22)] | 20 | 3 /20 (16%) | NA | NA | NA | NA | NA | NA | NA | NA | NA | NA |
| Smith[[25](#_ENREF_25)] | 37 | 11/23 (47%) | NA | NA | NA | NA | NA | NA | NA | NA | NA | NA |
| Teles Abrao Trad[[27](#_ENREF_27)] | 118 | NA | 10 /118 (8.5%) | NA | NA | NA | NA | NA | NA | NA | NA | NA |
| Thomas[[28](#_ENREF_28)] | 160 | NA | NA | NA | NA | 1/13 (1%) | NA | 9/13 (5%) | NA | NA | NA | NA |
| Trevisanuto[[53](#_ENREF_53)] | 44* | 12/38 (31.5%) | NA | NA | 7/34 (20%) | 9/34 (26%) | 17/34 (50%) | 3/34 (9%) | 15/21 (71%) | 41/44 (93%) | 3/44 (7%) | NA |
| Trippella[[29](#_ENREF_29)] | 248 | NA | 21 /189 (11.1%) | 1/189 (0.6%) | 4/160 (2.5%) | 13 /160 (8.1%) | 13 /160 (8.1%) | NA | 6/70 (8.6%) | 7/77 (44%) | NA | NA |
| Trocado[[30](#_ENREF_30)] | NA | NA | NA | NA | NA | NA | NA | NA | NA | 1/48 (2%) | NA | NA |
| Turan[[31](#_ENREF_31)] | 479 | NA | 50 /479 (10%) | NA | NA | NA | NA | NA | NA | 8 /479 (1.7%) | NA | NA |
| Yang N[[54](#_ENREF_54)] | 84 | NA | NA | NA | NA | NA | NA | NA | NA | NA | 4/84 (4.8%) | 7/84(8.3%) |
| Yang Z[[55](#_ENREF_55)] | 83 | NA | NA | NA | NA | NA | NA | NA | NA | 5 / 154 (1.8%) | NA | NA |
| Yee[[34](#_ENREF_34)] | 201 | 68 /73 (93.2%) | 8 /125 (6.4%) | NA | NA | 9 /125 (7.2%) | 5 /125 (4%) | 12 /125 (9.6%) | 18 /68 (26.5%) | 4 /167 (2.4%) | 3 /17 (17.6) | 6 /17 (35.3) |
| Yoon | NA | NA | NA | NA | NA | NA | NA | NA | NA | 1 /75 (1.3%) | NA | NA |

## ***Table 5: Neonatal outcomes in neonates born to SARS COV2 pregnant women***

| Author | N neonates | Neonatal birth weigth | Low birth weight (rate) | Small for Gestational age | Low Apgar (<7) | Preterm (<37 wks) | Congenital anomaly | Admission to NICU | Mechanical Ventilation | Mortality |
| --- | --- | --- | --- | --- | --- | --- | --- | --- | --- | --- |
| Akhtar[[2](#_ENREF_2)] | 108 | NA | NA | NA | NA | 27/108 (25%) | NA | NA | NA | 10/108 (9.2%) |
| Allotey[[3](#_ENREF_3)] | 2557 | NA | NA | NA | NA | 318/1872 (17%) | NA | 368/1348 (25%) | NA | 6/ 1728 (0.34%) |
| Arabi[[4](#_ENREF_4)] | 50 | 3200 g mean | NA | NA | NA | 10/50 (20%) | NA | NA | NA | 0/50 (0%) |
| Ashraf[[5](#_ENREF_5)] | 86 | 1520 - 3820 (range) | NA | 2/86 (2.3%) | NA | NA | NA | NA | NA | 1/86 (1.2%) |
| Banaei[[40](#_ENREF_40)] | 123 | 1580 - 4000 g (range) | NA | 2/123 (1.6%) | 1/123 (0.8%) | 30/123 (24.4%) | NA | 3/123 (2.4%) | NA | 1/123 (0.8) |
| Caparros Gonzalez *[[50](#_ENREF_50)] | 65 | NA | NA | 2/10 (20%) | NA | 6/10 (60%) | NA | NA | NA | 1/65 (1.53%) |
| Chi[[41](#_ENREF_41)] | 105 | NA | NA | 10 (11.2%) | NA | 25(23.8%) | NA | NA | NA | 1/105 (0.9%) |
| Della Gatta[[7](#_ENREF_7)] | 48 | NA | NA | NA | NA | 1/48 (2%) | NA | 1/48 (2%) | NA | 1/48 (2%) |
| Deniz[[42](#_ENREF_42)] | 606 | NA | NA | NA | NA | 44/606 (7.2%) | NA | NA | NA | NA |
| Dhir[[43](#_ENREF_43)] | 1141 | NA | NA | NA | NA | 281(25%) | NA | NA | NA | 0/58 (0%) |
| DiMascio[[44](#_ENREF_44)] | 41 | NA | NA | NA | 1/41 (2.4%) | 14/32 (43.8%) | NA | 1/10 (10%) | NA | 1/41 (2.4%) |
| Diriba[[8](#_ENREF_8)] | 1271 | NA | NA | NA | 1/72 (1.4%) | 369/686 (53.8%) | NA | 8/69 (11.6%) | NA | 5/430 (1.2%) |
| Duran[[51](#_ENREF_51)] | 222 | NA | NA | NA | 1/222 (0.45%) | 19/222 (8.5%) | NA | NA | NA | 1/222 (0.45%) |
| Figueiro-Filho[[9](#_ENREF_9)] | Depends on each outcome | NA | 28/259 (11%) | NA | NA | 159/764 (21%) | 8/241 (3.3%) | 183/992 (18.45%) | NA | 9/1130 (0.8%) |
| Furlan[[10](#_ENREF_10)] | NA | NA | NA | NA | NA | NA | NA | NA | NA | 1/189 (0.5%) |
| Gajbhiye[[11](#_ENREF_11)] | 391 | NA | NA | NA | NA | 98/386 (26%) | NA | 31/391 (8%) | NA | 4/369 (1.6 %) |
| Gao[[12](#_ENREF_12)] | NA | NA | NA | NA | NA | 26/116 (23%) | NA | NA | NA | 1/147 (0.7%) |
| Han[[13](#_ENREF_13)] | NA | NA | 12/33(30.65%) | NA | 12/160 (18.76%) | 118/743(25.3%) | NA | 108/341(24.4%) | 7/54 (11.1%) | 5/599 (0.8%) |
| Huntley[[14](#_ENREF_14)] | NA | NA | NA | NA | 1/203 (0.5%) | 57/284 (20.1%) | NA | 137/211 (64.9%) | NA | 1/313 (0.3%) |
| Juan[[15](#_ENREF_15)] | 221 | NA | NA | NA | NA | NA | NA | 49/173 (28.3%) | NA | 1/221 (0.5%) |
| Kasraeian[[16](#_ENREF_16)] | Depends on each outcome | NA | NA | NA | NA | 25/41 (60.9%) | NA | NA | NA | 1/86 (0.2%) |
| Khalil[[17](#_ENREF_17)] | 598 | NA | 45/598 (7.5%) | NA | NA | 198/598 (33.2%) | NA | 179/598 (30%) | NA | 2/528(0.4%) |
| Matar[[20](#_ENREF_20)] | 136 | 3127.64 g mean | NA | NA | NA | 31/94 (33%) | NA | 27/42 (63.7%) | NA | 3/94 (3.2%) |
| Mirbeyk[[21](#_ENREF_21)] | 302 | NA | NA | NA | NA | 65 /302 (21.5%) | NA | 5/302 (1.6%) | NA | 2/302 (0.7%) |
| Muhidin[[47](#_ENREF_47)] | 89 | 1520 to 3820 g range | 7/89 (7.9%) | 2/89 (2.2%) | NA | NA | NA | NA | NA | 2/89 (2.2%) |
| Mullins[[22](#_ENREF_22)] | 20 | NA | NA | NA | NA | 8/19 (42%) | NA | 1/20 (5%) | NA | 1/20 (5%) |
| Panahi[[56](#_ENREF_56)] | ** | NA | NA | NA | NA | NA | NA | NA | NA | 1/10 (10%)* |
| Rodriguez Blanco [[48](#_ENREF_48)] | 74 | NA | NA | NA | 1/74 (1.3%) | NA | NA | NA | NA | 1/74 (1.3%) |
| Segars[[49](#_ENREF_49)] | 447 | NA | 37/244(15.2%) | NA | 1/17 (5.9%) | 128/236 (54.2%) | NA | NA | NA | 0/91 (0%) |
| Smith[[25](#_ENREF_25)] | 37 | 2743 g (mean) | 9/21 (42.9 %) | NA | 0/32 (0%) | 6/13 (68.8%) | NA | 11/of 13 (76.9%) | NA | 1/36 (2.7%) |
| Soheili[[26](#_ENREF_26)] | NA | NA | 13/63 (21%) | NA | NA | 42/151 (28%) | NA | NA | NA | 2/37 (4%) |
| Teles Abrao Trad[[27](#_ENREF_27)] | 118 | NA | 14/118 ( 11.8%) | NA | NA | 19/118 (16.1%)******** | NA | 24/118 (20.3%) | NA | 1/118 (0.8%) |
| Thomas [[28](#_ENREF_28)] | 160 | NA | NA | 3/47 (7%) | NA | 24/118 /20%) | NA | NA | NA | 1/160(1%) |
| Trevisanuto[[53](#_ENREF_53)] | 44 *** | NA | NA | NA | NA | NA | NA | 6/36 (16.6%) | NA | 0/44 (0%) |
| Tripella[[29](#_ENREF_29)] | 248 | 2914 grams (mean) | NA | NA | 5/190 | 54/196 (27.5%) | NA | NA | NA | 1/248 (0.4%) |
| Trocado[[30](#_ENREF_30)] | NA | 2292 grams (mean) | 10 (20%) | NA | NA | 18 (35%) | NA | NA | NA | 1/51 (2%) |
| Turan[[31](#_ENREF_31)] | 479 | NA | NA | 6/479 (1.25%) | 6/361 (1.66%) | 161/479 (24.8%) | NA | 54/479 (11.3%) | NA | 5/479 (1%) |
| Yang N[[54](#_ENREF_54)] | 84 | NA | 2/38 (5.3%) | NA | NA | 17/80 (21.3%) | NA | NA | NA | 1/84 (1.2%) |
| Yee[[34](#_ENREF_34)] | 190 | 2855.9 grams (mean) | NA | 9/64 (17.4%) | NA | 54/190 (28.6%) | NA | NA | NA | 1/103 (0.4%) |
| Yoon[[35](#_ENREF_35)] | 201 | 1880 g to 4050 g range | 15/96 (15.6%) | 5/60 (8.3%) | NA | 48/185 (25.9%) | NA | NA | NA | 1/177 (0.6%) |
| Zaigham[[36](#_ENREF_36)] | NA | NA | NA | NA | NA | NA | NA | NA | NA | 1/87 (1%) |

* Zhu et al.

** Involves neonates and children up to 16 years/age (it cannot be determined how many neonates are)

***neonates with SARS-CoV-2 infection

**** <36 weeks

## ***Table 6: Vertical transmission to neonates born to SARS COV2 mothers***

| Authors (2020) | N pregnant women | N neonates | Number of sars-cov-2 cases in neonates | Congenital or perinatal | Breastfeeding or breast milk | Respiratory droplets |
| --- | --- | --- | --- | --- | --- | --- |
| AbdelMassih[[1](#_ENREF_1)] | 1787 | 1787 | 49 | NA | NA | 49 |
| Abdollahpour[[39](#_ENREF_39)] | NA | NA | 2 | NA | 0/6 (0%) | NA |
| Ashraf[[5](#_ENREF_5)] |  | 86 | 4 | 0/6 (0%) Placenta  1/16 (6.25%) Amniotic | NA | 4/86 (4.7%) |
| Banaei[[40](#_ENREF_40)] | NA | NA | 5 | NA | NA | NA |
| Bwire[[57](#_ENREF_57)] | NA | 206 | 13/206 (6,3%) | NA | NA | NA |
| Caparros Gonzalez[[50](#_ENREF_50)] | NA | NA | 0/13 (0%) | NA | NA | NA |
| Centeno-Tablante[[58](#_ENREF_58)] |  | 889 | 124 | NA | 14/82 (17%) | 124/889 (13.9%) |
| Chi[[41](#_ENREF_41)] | NA | 91 | 8 | NA | NA | NA |
| Deniz[[42](#_ENREF_42)] | NA | 606 | 20 | 8/63 (12.7%) Placenta  1/8 (12.5%) Amniotic | 3/6 (50%) | NA |
| De Sousa[[6](#_ENREF_6)] | NA | 493 | 9 | 0/54 (0%) Placenta | NA | NA |
| Dhir[[43](#_ENREF_43)] | 43 | 148 | 58 | 4/58 (6.9%) Amniotic | NA | 41/58 (70.7%) |
| Di Mascio[[44](#_ENREF_44)] | NA | 42 | 0/42 (0%) | NA | NA | NA |
| Diriba[[8](#_ENREF_8)] | 1271 | 1271 | 0/1271 (0%) | NA | NA | NA |
| Duran[[51](#_ENREF_51)] | NA | 222 | 13 | NA | NA | NA |
| Figueiro-Filho[[9](#_ENREF_9)] | NA | 1116 | 18/1116 (1,5%) | NA | NA | NA |
| Furlan[[10](#_ENREF_10)] | NA | 188 | 4 | 0 | NA | NA |
| Gajbhiye[[11](#_ENREF_11)] | 387 | 313 | 24 | NA | NA | 24/313 (7.7%) |
| Goh[[59](#_ENREF_59)] | NA | 330 | 9/33 (27.3%) | NA | NA | NA |
| Gordon[[52](#_ENREF_52)] | NA | 46 | 10 | 0/1 (0%) | NA | 10/46 (21.7%) |
| Han[[13](#_ENREF_13)] | NA | 559 | 21 | 1/13 (7.7%) Placenta  0/16 (0%) Cord blood  0/17 (0%) Amniotic fluid | 0/10 (0%) | 21/559 (3.8%) |
| Hasan[[60](#_ENREF_60)] | NA | NA | 0 | NA | NA | NA |
| Hessami[[61](#_ENREF_61)] | NA | 310 | 0 | NA | NA | NA |
| Juan[[15](#_ENREF_15)] | NA | 155 | 3/90 (3.3%) | 1/32 (3.1%) Amniotic  0/34 (0%) Cord blood  1/3 (33%) Placenta | 0/22 (0%) | 3/155 (1.9%) |
| Khalil[[17](#_ENREF_17)] | NA | 751 | 19 | 3/38 (7.9%) Placenta  1/18 (5.6%) Amniotic  1/16 (6.3%) Cord blood | 2/30 (6,6%) | NA |
| Kotlyar[[62](#_ENREF_62)] | NA | 936 | 27/843 (3.2%) | 2/26 (7.7%) Placenta  0/51 (0%) Amniotic | 2/47 (4,2%) | 27/843 (3,2%) |
| Martins[[45](#_ENREF_45)] | 24 | 24 | 0 | 0/22 (0%) | 0/24 (0%) | 0/22 (0%) |
| Matar[[20](#_ENREF_20)] | 136 | 24 | 2/17 (11.5% [95% CI, .067–.192]; I^2^ = 0). | 0/24 (0%) Amniotic  0/24 (0%) Placenta  0/24 (0%) Cord blood. | NA | NA |
| Melo[[46](#_ENREF_46)] | NA | 405 | 10 | 0/45 (0%) Amniotic  3/26 (11.5%) Placenta  4/28 (14.3%) Cord blood | 0/44 (0%) | 10/405 (2,5%) |
| Mirbeyk[[21](#_ENREF_21)] | NA | 219 | 11 | 0/219 (0%) Cord blood  0/219 (0%) Placenta  1/219 (0.4%) (Amniotic | 0 | NA |
| Mullins[[22](#_ENREF_22)] | NA | 15 | 0 | NA | NA | NA |
| Mustafa[[63](#_ENREF_63)] | NA | 57 | 3 | 0/11 (0%) Amniotic  0/11 (0%) Cord blood  0/2 (0%) Placenta | 0/11 (0%) | 3/57 (5,3%) |
| Pettirosso[[23](#_ENREF_23)] | NA | 655 | 19 | 4/19 (21%) Placenta  1/19 (5.3%)Cord blood | 0/45 (0%) | 19/655 (2,9%) |
| Rodrí­guez-Blanco[[48](#_ENREF_48)] | NA | 66 | 0/66 (0%) | NA | 0/6 (0%) | NA |
| Singh[[64](#_ENREF_64)] | 62 | NA | NA | 2/27 (7,4%) Placenta. | NA | NA |
| Smith[[25](#_ENREF_25)] | NA | 37 | 1/37 (2.7%) | 0/9 (0%) Cord blood  0/9 (0%) Amniotic | NA | 1/37 (2.7%) |
| Teles Abrao Trad[[27](#_ENREF_27)] | NA | 95 | 1/95 (1%) | 0 | 0 | 1/95 (1%) |
| Thomas[[28](#_ENREF_28)] | NA | 81 | 5 | 9/81 (11,1%) Cord blood  9/81 (11,1%) Amniotic | 16/81 (19,8%) | NA |
| Trippella[[29](#_ENREF_29)] | NA | 191 | 16 | 0/35 (0%) | 0/25 (0%) | 16/191 (8,4%) |
| Turan[[31](#_ENREF_31)] | 637 | 479 | 8/400 (2%) | 0/20 (0%) Amniotic  0/19 (0%) Cord blood  1/6 (16,7%) Placenta | 1/19 (5,26%) | 8/405 (2%) |
| Yang N[[54](#_ENREF_54)] | 114 | NA | 2/74 (2,7%) | 0/19 (0%) | 0/17 (0%) | 2/59 (3,4%) |
| Yang Z[[55](#_ENREF_55)] | NA | 83 | 3/83 (3,6%) | 0/18 (0%) | 0/14 (0%) | 3/62 (4,8%) |
| Yang Z[[33](#_ENREF_33)] | 16 | 12 | 1 | NA | 0/16 (0%) | 1/12 (8,3%) |
| Yee[[34](#_ENREF_34)] | NA | 154 | 5 (1,8%) | NA | NA | NA |
| Yoon[[35](#_ENREF_35)] | 4 | 4 | 4 | 0/4 (0%) Cord blood  0/3 (0%) Amniotic fluid  0/1 (0%) Placenta | 0/4 (0%) | 4/4 (100%) |
| Zaigham[[36](#_ENREF_36)] | 108 | 75 | 1/75 (1%) | NA | NA | NA |
| Rahman[[24](#_ENREF_24)] | NA | NA | Narrative description: cases of vertical COVID-19 transmission are few and maybe incidental, the potential of vertical transmission of COVID19 should not be ruled out. | NA | NA | NA |

**References**

1. AbdelMassih A, Fouda R, Essam R, Negm A, Khalil D, Habib D, et al. COVID-19 during pregnancy should we really worry from vertical transmission or rather from fetal hypoxia and placental insufficiency? A systematic review and meta -analysis. 2020. doi: 10.21203/rs.3.rs-71847/v1.

2. Akhtar H, Patel C, Abuelgasim E, Harky A. COVID-19 (SARS-CoV-2) Infection in Pregnancy: A Systematic Review. Gynecologic and Obstetric Investigation. 2020. doi: 10.1159/000509290.

3. Allotey J, Stallings E, Bonet M, Yap M, Chatterjee S, Kew T, et al. Clinical manifestations, risk factors, and maternal and perinatal outcomes of coronavirus disease 2019 in pregnancy: living systematic review and meta-analysis. BMJ (Clinical research ed). 2020;370:m3320. doi: 10.1136/bmj.m3320.

4. Arabi S, Vaseghi G, Heidari Z, Shariati L, Amin B, Rashid H, et al. Clinical characteristics of COVID-19 infection in pregnant women: a systematic review and meta-analysis. medRxiv. 2020:2020.04.05.20053983. doi: 10.1101/2020.04.05.20053983.

5. Ashraf MA, Keshavarz P, Hosseinpour P, Erfani A, Roshanshad A, Pourdast A, et al. Coronavirus disease 2019 (COVID-19): A systematic review of pregnancy and the possibility of vertical transmission. Journal of Reproduction and Infertility. 2020;21(3):157-68.

6. de Sousa AFL, de Carvalho HEF, de Oliveira LB, Schneider G, Camargo ELS, Watanabe E, et al. Effects of COVID-19 Infection during Pregnancy and Neonatal Prognosis: What Is the Evidence? Int J Environ Res Public Health. 2020;17(11):17. doi: 10.3390/ijerph17114176. PubMed PMID: WOS:000542629600429.

7. Della Gatta AN, Rizzo R, Pilu G, Simonazzi G. Coronavirus disease 2019 during pregnancy: a systematic review of reported cases. American Journal of Obstetrics and Gynecology. 2020;223(1):36-41. doi: 10.1016/j.ajog.2020.04.013.

8. Diriba K, Awulachew E, Getu E. The effect of coronavirus infection (SARS-CoV-2, MERS-CoV, and SARS-CoV) during pregnancy and the possibility of vertical maternal-fetal transmission: a systematic review and meta-analysis. European journal of medical research. 2020;25(1):39. doi: 10.1186/s40001-020-00439-w.

9. Figueiro-Filho EA, Yudin M, Farine D. COVID-19 during pregnancy: an overview of maternal characteristics, clinical symptoms, maternal and neonatal outcomes of 10,996 cases described in 15 countries. J Perinat Med. 2020. doi: 10.1515/jpm-2020-0364. PubMed PMID: 33001856.

10. Furlan MCR, Jurado SR, Uliana CH, Silva MEP, Nagata LA, Maia ACF. Gravidez e infecção por Coronavírus: desfechos maternos, fetais e neonatais ­ Revisão sistemática^iptA Systematic Review of Pregnancy and Coronavirus Infection: Maternal, Fetal and Neonatal Outcomes^ienRevisión sistemática del embarazo y la infección por. rev cuid (Bucaramanga 2010). 2020;11(2).

11. Gajbhiye R, Modi D, Mahale S. Pregnancy outcomes, Newborn complications and Maternal-Fetal Transmission of SARS-CoV-2 in women with COVID-19: A systematic review of 441 cases. medRxiv. 2020:2020.04.11.20062356. doi: 10.1101/2020.04.11.20062356.

12. Gao YJ, Ye L, Zhang JS, Yin YX, Liu M, Yu HB, et al. Clinical features and outcomes of pregnant women with COVID-19: A systematic review and meta-analysis. BMC Infectious Diseases. 2020;20(1). doi: 10.1186/s12879-020-05274-2.

13. Han Y, Ma H, Suo M, Han F, Wang F, Ji J, et al. Clinical manifestation, outcomes in pregnant women with COVID-19 and the possibility of vertical transmission: a systematic review of the current data. J Perinat Med. 2020. doi: 10.1515/jpm-2020-0431. PubMed PMID: 33068387.

14. Huntley BJF, Huntley ES, Di Mascio D, Chen T, Berghella V, Chauhan SP. Rates of Maternal and Perinatal Mortality and Vertical Transmission in Pregnancies Complicated by Severe Acute Respiratory Syndrome Coronavirus 2 (SARS-Co-V-2) Infection: A Systematic Review. Obstetrics and gynecology. 2020;136(2):303-12. doi: 10.1097/AOG.0000000000004010.

15. Juan J, Gil MM, Rong Z, Zhang Y, Yang H, Poon LC. Effect of coronavirus disease 2019 (COVID-19) on maternal, perinatal and neonatal outcome: systematic review. Ultrasound in obstetrics & gynecology : the official journal of the International Society of Ultrasound in Obstetrics and Gynecology. 2020;56(1):15-27. doi: 10.1002/uog.22088.

16. Kasraeian M, Zare M, Vafaei H, Asadi N, Faraji A, Bazrafshan K, et al. COVID-19 pneumonia and pregnancy; a systematic review and meta-analysis. Journal of Maternal-Fetal and Neonatal Medicine. 2020. doi: 10.1080/14767058.2020.1763952.

17. Khalil A, Kalafat E, Benlioglu C, O'Brien P, Morris E, Draycott T, et al. SARS-CoV-2 infection in pregnancy: A systematic review and meta-analysis of clinical features and pregnancy outcomes. EClinicalMedicine. 2020;25. doi: 10.1016/j.eclinm.2020.100446.

18. Khan MMA, Khan MN, Mustagir MG, Rana J, Haque MR, Rahman MM. COVID-19 infection during pregnancy: A systematic review to summarize possible symptoms, treatments, and pregnancy outcomes. Cold Spring Harbor Laboratory; 2020.

19. Li W, Tang J, Zeng Y, Yue Y, He Y, Zhang M, et al. A systematic review of SARS-infected pregnant females, newborns, children and adolescents. Chinese Journal Of Evidence-Based Medicine. 2020;20(04):426-36.

20. Matar R, Alrahmani L, Monzer N, Debiane LG, Berbari E, Fares J, et al. Clinical Presentation and Outcomes of Pregnant Women with COVID-19: A Systematic Review and Meta-Analysis. Clinical infectious diseases : an official publication of the Infectious Diseases Society of America. 2020. doi: 10.1093/cid/ciaa828.

21. Mirbeyk M, Rezaei N. The impact of COVID-19 on pregnancy and neonatal health: a systematic review. Research Square; 2020.

22. Mullins E, Evans D, Viner R, O'Brien P, Morris E. Coronavirus in pregnancy and delivery: rapid review and expert consensus. Cold Spring Harbor Laboratory; 2020.

23. Pettirosso E, Giles M, Cole S, Rees M. COVID-19 and pregnancy: A review of clinical characteristics, obstetric outcomes and vertical transmission. Australian and New Zealand Journal of Obstetrics and Gynaecology. 2020. doi: 10.1111/ajo.13204.

24. Rahman HS, Aziz MS, Hussein RH, Othman HH, Salih Omer SH, Khalid ES, et al. The transmission modes and sources of COVID-19: A systematic review. International Journal of Surgery Open. 2020;26:125-36. doi: 10.1016/j.ijso.2020.08.017.

25. Smith V, Seo D, Warty R, Payne O, Salih M, Chin KL, et al. Maternal and neonatal outcomes associated with COVID-19 infection: A systematic review. PLoS ONE. 2020;15(6). doi: 10.1371/journal.pone.0234187.

26. Soheili M, Moradi G, Baradaran HR, Soheili M, Moradi Y. Clinical Manifestation and Maternal Complications and Neonatal outcomes in Pregnant Women with COVID 19: An Update a Systematic Review and Meta-analysis. Research Square; 2020.

27. Teles Abrao Trad A, Ibirogba ER, Elrefaei A, Narang K, Tonni G, Picone O, et al. Complications and outcomes of SARS-CoV-2 in pregnancy: where and what is the evidence? Hypertension in pregnancy. 2020;39(3):361-9. doi: 10.1080/10641955.2020.1769645. PubMed PMID: 32456489.

28. Thomas P, Alexander PE, Ahmed U, Elderhorst E, El-Khechen H, Mammen MJ, et al. Vertical transmission risk of SARS-CoV-2 infection in the third trimester: a systematic scoping review. Journal of Maternal-Fetal and Neonatal Medicine. 2020. doi: 10.1080/14767058.2020.1786055.

29. Trippella G, Ciarcia M, Ferrari M, Buzzatti C, Maccora I, Azzari C, et al. COVID-19 in Pregnant Women and Neonates: A Systematic Review of the Literature with Quality Assessment of the Studies. Pathogens. 2020;9(6):25. doi: 10.3390/pathogens9060485. PubMed PMID: WOS:000551563600001.

30. Trocado V, Silvestre-Machado J, Azevedo L, Miranda A, Nogueira-Silva C. Pregnancy and COVID-19: a systematic review of maternal, obstetric and neonatal outcomes. Journal of Maternal-Fetal and Neonatal Medicine. 2020:1-13. doi: 10.1080/14767058.2020.1781809.

31. Turan O, Hakim A, Dashraath P, Jeslyn WJL, Wright A, Abdul-Kadir R. Clinical characteristics, prognostic factors, and maternal and neonatal outcomes of SARS-CoV-2 infection among hospitalized pregnant women: A systematic review. International Journal of Gynecology and Obstetrics. 2020;151(1):7-16. doi: 10.1002/ijgo.13329.

32. Uygun-Can B, Acar-Bolat B. Clinical Properties and Diagnostic Methods of COVID-19 Infection in Pregnancies: Meta-Analysis. BioMed Research International. 2020:1-8. doi: 10.1155/2020/1708267. PubMed PMID: 146144524. Language: English. Entry Date: In Process. Revision Date: 20201001. Publication Type: Article. Journal Subset: Biomedical.

33. Yang Z, Wang M, Zhu Z, Liu Y. Coronavirus disease 2019 (COVID-19) and pregnancy: a systematic review. Journal of Maternal-Fetal and Neonatal Medicine. 2020. doi: 10.1080/14767058.2020.1759541.

34. Yee J, Kim W, Han JM, Yoon HY, Lee N, Lee KE, et al. Clinical manifestations and perinatal outcomes of pregnant women with COVID-19: a systematic review and meta-analysis. Sci Rep. 2020;10(1):18126. doi: 10.1038/s41598-020-75096-4. PubMed PMID: 33093582.

35. Yoon SH, Kang JM, Ahn JG. Clinical outcomes of 201 neonates born to mothers with COVID-19: A systematic review. European Review for Medical and Pharmacological Sciences. 2020;24(14):7804-15. doi: 10.26355/eurrev_202007_22285.

36. Zaigham M, Andersson O. Maternal and perinatal outcomes with COVID-19: A systematic review of 108 pregnancies. Acta Obstetricia et Gynecologica Scandinavica. 2020;99(7):823-9. doi: 10.1111/aogs.13867.

37. Shi L, Wang Y, Yang H, Duan G. Laboratory Abnormalities in Pregnant Women with Novel Coronavirus Disease 2019. American journal of perinatology. 2020;37(10):1070-3. doi: 10.1055/s-0040-1712181. PubMed PMID: 32396949.

38. Vakili S, Savardashtaki A, Jamalnia S, Tabrizi R, Nematollahi MH, Jafarinia M, et al. Laboratory Findings of COVID-19 Infection are Conflicting in Different Age Groups and Pregnant Women: A Literature Review. Archives of Medical Research. 2020. doi: 10.1016/j.arcmed.2020.06.007.

39. Abdollahpour S, Khadivzadeh T. Improving the quality of care in pregnancy and childbirth with coronavirus (COVID-19): a systematic review. J Matern-Fetal Neonatal Med. 2020:9. doi: 10.1080/14767058.2020.1759540. PubMed PMID: WOS:000534953600001.

40. Banaei M, Ghasemi V, Saei M, Naz MSG, Kiani Z, Rashidi-Fakari F, et al. Obstetrics and Neonatal Outcomes in Pregnant Women with COVID-19: A Systematic Review. Iran J Public Health. 2020;49:38-47. PubMed PMID: WOS:000531776100006.

41. Chi H, Chiu NC, Tai YL, Chang HY, Lin CH, Sung YH, et al. Clinical features of neonates born to mothers with coronavirus disease-2019: A systematic review of 105 neonates. Journal of microbiology, immunology, and infection = Wei mian yu gan ran za zhi. 2020. doi: 10.1016/j.jmii.2020.07.024. PubMed PMID: 32847748.

42. Deniz M, Tezer H. Vertical transmission of SARS CoV-2: a systematic review. Journal of Maternal-Fetal and Neonatal Medicine. 2020:1-8. doi: 10.1080/14767058.2020.1793322.

43. Dhir SK, Kumar J, Meena J, Kumar P. Clinical Features and Outcome of SARS-CoV-2 Infection in Neonates: A Systematic Review. Journal of tropical pediatrics. 2020. doi: 10.1093/tropej/fmaa059.

44. Di Mascio D, Khalil A, Saccone G, Rizzo G, Buca D, Liberati M, et al. Outcome of Coronavirus spectrum infections (SARS, MERS, COVID 1 -19) during pregnancy: a systematic review and meta-analysis. American Journal of Obstetrics & Gynecology MFM. 2020;2(2):100107. doi: 10.1016/j.ajogmf.2020.100107.

45. Martins PR, Santos VS, Santos HP. To breastfeed or not to breastfeed? Lack of evidence on the presence of SARS-CoV-2 in breastmilk of pregnant women with COVID-19. Rev Panam Salud Publica. 2020;44:7. doi: 10.26633/rpsp.2020.59. PubMed PMID: WOS:000529448200001.

46. Melo GC, Araújo K. COVID-19 infection in pregnant women, preterm delivery, birth weight, and vertical transmission: a systematic review and meta-analysis. Cadernos de saude publica. 2020;36(7):e00087320. doi: 10.1590/0102-311x00087320. PubMed PMID: 32696830.

47. Muhidin S, Behboodi Moghadam Z, Vizheh M. Analysis of Maternal Coronavirus Infections and Neonates Born to Mothers with 2019-nCoV; a Systematic Review. Archives of academic emergency medicine. 2020;8(1):e49. doi: 10.22037/AAEM.V8I1.656.G788.

48. Rodríguez-Blanco N, Vegara-Lopez I, Aleo-Giner L, Tuells J. [Scoping review of coronavirus case series (SARS-CoV, MERS-CoV and SARS-CoV-2) and their obstetric and neonatal results]. Revista espanola de quimioterapia : publicacion oficial de la Sociedad Espanola de Quimioterapia. 2020. doi: 10.37201/req/064.2020. PubMed PMID: 32683837.

49. Segars J, Katler Q, McQueen DB, Kotlyar A, Glenn T, Knight Z, et al. Prior and novel coronaviruses, Coronavirus Disease 2019 (COVID-19), and human reproduction: what is known? Fertility and sterility. 2020;113(6):1140-9. doi: 10.1016/j.fertnstert.2020.04.025. PubMed PMID: 32482250.

50. CaparrosGonzalez Rafael A. Maternal and neonatal consequences of coronavirus COVID-19 infection during pregnancy: a scoping review. Revista espanola de salud publica. 2020.

51. Duran P, Berman S, Niermeyer S, Jaenisch T, Forster T, Ponce de Leon RG, et al. COVID-19 and newborn health: systematic review^ien. Rev panam salud pública. 2020;44.

52. Gordon M, Kagalwala T, Rezk K, Rawlingson C, Ahmed MI, Guleri A. Rapid systematic review of neonatal COVID-19 including a case of presumed vertical transmission. BMJ Paediatrics Open. 2020;4(1). doi: 10.1136/bmjpo-2020-000718.

53. Trevisanuto D, Cavallin F, Cavicchiolo ME, Borellini M, Calgaro S, Baraldi E. Coronavirus infection in neonates: A systematic review. Archives of Disease in Childhood: Fetal and Neonatal Edition. 2020. doi: 10.1136/archdischild-2020-319837.

54. Yang N, Che S, Zhang J, Wang X, Tang Y, Wang J, et al. Breastfeeding of infants born to mothers with COVID-19: A rapid review. Annals of Translational Medicine. 2020;8(10). doi: 10.21037/atm-20-3299.

55. Yang Z, Liu Y. Vertical Transmission of Severe Acute Respiratory Syndrome Coronavirus 2: A Systematic Review. American Journal of Perinatology. 2020;37(1):1055-60. doi: 10.1055/s-0040-1712161.

56. Panahi L, Amiri M, Pouy S. Clinical Characteristics of COVID-19 Infection in Newborns and Pediatrics: A Systematic Review. Archives of academic emergency medicine. 2020;8(1):e50. PubMed PMID: 32440661.

57. Bwire GM, Njiro BJ, Mwakawanga DL, Sabas D, Sunguya BF. Possible vertical transmission and antibodies against SARS-CoV-2 among infants born to mothers with COVID-19: A living systematic review. J Med Virol. 2020. doi: 10.1002/jmv.26622. PubMed PMID: 33090535.

58. Centeno-Tablante E, Medina-Rivera M, Finkelstein JL, Rayco-Solon P, Garcia-Casal MN, Rogers L, et al. Transmission of SARS-CoV-2 through breast milk and breastfeeding: a living systematic review. Annals of the New York Academy of Sciences. 2020. doi: 10.1111/nyas.14477.

59. Goh XL, Low YF, Ng CH, Amin Z, Ng YPM. Incidence of SARS-CoV-2 vertical transmission: a meta-analysis. Archives of disease in childhood Fetal and neonatal edition. 2020. doi: 10.1136/archdischild-2020-319791.

60. Hasan MZ, Kibria GMA, Alam T. Pregnancy during the evolving pandemic Coronavirus Disease 2019 (COVID-19): A rapid scoping review of early evidence in the published literature. Research Square; 2020.

61. Hessami K, Homayoon N, Hashemi A, Vafaei H, Kasraeian M, Asadi N. COVID-19 and maternal, fetal and neonatal mortality: a systematic review. Journal of Maternal-Fetal and Neonatal Medicine. 2020. doi: 10.1080/14767058.2020.1806817.

62. Kotlyar AM, Grechukhina O, Chen A, Popkhadze S, Grimshaw A, Tal O, et al. Vertical transmission of coronavirus disease 2019: a systematic review and meta-analysis. American Journal of Obstetrics and Gynecology. 2020. doi: 10.1016/j.ajog.2020.07.049.

63. Mustafa NM, L AS. Characterisation of COVID-19 Pandemic in Paediatric Age Group: A Systematic Review and Meta-Analysis. Journal of clinical virology : the official publication of the Pan American Society for Clinical Virology. 2020;128:104395. doi: 10.1016/j.jcv.2020.104395. PubMed PMID: 32417675.

64. Singh B, Gornet M, Sims H, Kisanga E, Knight Z, Segars J. Severe Acute Respiratory Syndrome-Corona Virus-2 (SARS-CoV-2) and its Effect on Gametogenesis and Early Pregnancy. Am J Reprod Immunol. 2020:e13351. doi: 10.1111/aji.13351. PubMed PMID: 32969123.
